# Development of Three Sets of High-Throughput Genotyped Rice Chromosome Segment Substitution Lines and QTL Mapping for Eleven Traits

**DOI:** 10.1186/s12284-019-0293-y

**Published:** 2019-05-10

**Authors:** Bin Zhang, Lianguang Shang, Banpu Ruan, Anpeng Zhang, Shenglong Yang, Hongzhen Jiang, Chaolei Liu, Kai Hong, Hai Lin, Zhenyu Gao, Jiang Hu, Dali Zeng, Longbiao Guo, Qian Qian

**Affiliations:** 1 State Key Laboratory of Rice Biology, China National Rice Research Institute, Chinese Academy of Agricultural Sciences, Hangzhou, 310006 China; 2 Agricultural Genomics Institute at Shenzhen, Chinese Academy of Agricultural Sciences, Shenzhen, 518124 China

**Keywords:** Chromosome segment substitution lines (CSSLs), High-throughput resequencing, QTL mapping, Grain length, GWAS

## Abstract

**Background:**

Detecting and mapping chromosomal regions that are related to quantitative phenotypic variation in chromosome segment substitution lines (CSSLs) provides an effective means to characterize the genetic basis of complex agronomic trait. CSSLs are also powerful tools for studying the effects of quantitative trait loci (QTLs) pyramiding and interaction on phenotypic variation.

**Results:**

Here, we developed three sets of CSSLs consisting of 81, 55, and 61 lines, which were derived from PA64s × 9311, Nipponbare × 9311 and PA64s × Nipponbare crosses, respectively. All of the 197 CSSLs were subjected to high-throughput genotyping by whole-genome resequencing to obtain accurate physical maps for the 3 sets of CSSLs. The 3 sets of CSSLs were used to analyze variation for 11 major agronomic traits in Hangzhou and Shenzhen and led to the detection of 71 QTLs with phenotypic effect that ranged from 7.6% to 44.8%. Eight QTLs were commonly detected under two environments for the same phenotype, and there were also 8 QTL clusters that were found. Combined with GWAS on grain length and expression profiles on young panicle tissues, *qGL1* detected in CSSLs was fine mapped within a 119 kb region on chromosome 1 and *LOC_Os01g53140* and *LOC_Os01g53250* were the two most likely candidate genes.

**Conclusions:**

Our results indicate that developing CSSLs genotyped by whole-genome resequencing are powerful tools for basic genetic research and provide a platform for the rational design of rice breeding. Meanwhile, the conjoint analysis of different CSSLs, natural population and expression profiles can facilitate QTL fine mapping.

**Electronic supplementary material:**

The online version of this article (10.1186/s12284-019-0293-y) contains supplementary material, which is available to authorized users.

## Background

Most important agronomic traits of rice (*Oryza sativa* L.), such as heading date, yield, plant height and grain size, are controlled by quantitative trait loci (QTLs). Although multiple QTLs have been reported over the past few decades, few of them have been isolated or fine-mapped (Hu et al. 2015; Huang et al. 2009a; Ishimaru et al. 2013; Jiao et al. 2010; Konishi et al. 2006; Song et al. 2007; Wang et al. 2015; Xue et al. 2008; Yano et al. 2000). As the phenotypic manifestations of these quantitative traits show continuous variation and are always influenced by environmental factors and genetic backgrounds, only suitable genetic populations can provide a strong foundation for genetic and genomic research. To date, many types of genetic populations have been developed for QTL research and utilization. Primary genetic mapping populations, such as the F_2,_ F_2:3_ and BC_1_ populations, were often used for analyzing the genetic basis of QTL during early studies (Ahn et al. 1993; Li et al. 1995; Redona and Mackill 1996). However, these types of genetic populations can only detect a few major QTLs with large genetic effects due to complex and unstable genetic backgrounds (Yamamoto et al. 2000). To further explore the genetic basis of complex traits, some permanent mapping populations, such as recombinant inbred lines (RILs) and doubled haploid (DH) have been developed. QTLs with minor effects can also be detected by using these permanent populations, but they are still inadequate for fine mapping and cloning of QTLs (Yano 2001). As a permanent population, chromosome segment substitution lines (CSSLs) can improve the precise detection of QTL regulation of complex traits and make it much easier for QTL fine mapping and cloning (Mei et al. 2006; Takai et al. 2007). Each line of CSSLs has the same genetic background as its recurrent parent, except for a few substituted segments from a donor parent. CSSLs are ideal for mapping genetic population for the elimination of most genetic background noise and dissection of QTLs into the single Mendelian factor (Xu et al. 2010). Although development of CSSLs is laborious and time-consuming, more and more CSSLs have been developed for their significant advantages (Bessho-Uehara et al. 2017; Furuta et al. 2014; Hao et al. 2006; Kubo et al. 2002; Qiao et al. 2016; Shim et al. 2010; Yoshimura 1997).

Marker-assisted selection (MAS) is a powerful technique of selecting target segments during CSSLs construction. With the publication of rice whole genome sequences, many SSR (simple sequence repeat) markers, Indels (insertions and deletions) markers and SNPs (single nucleotide polymorphisms) markers have been exploited for MAS. The density of molecular markers that are distributed in the whole genome determine the accuracy of CSSLs. Low-density molecular makers may easily miss small introgression segments and double-crossovers (Xu et al. 2010). In fact, less than 200 molecular markers were used to develop CSSLs because of laborious work (Bian et al. 2010; Shim et al. 2010; Xu et al. 2007; Zhu et al. 2009). Next-generation sequencing technology provides a high-throughput genotyping method that can construct high-resolution physical maps and calculate the length of substituted segments in the construction of CSSLs. Recently, several RILs and CSSLs have been exactly genotyped by whole-genome resequencing, and high-quality linkage maps were constructed for QTL dissection (Gao et al. 2013; Huang et al. 2009b; Xu et al. 2010; Zhang et al. 2011; Zhu et al. 2015b). Among the CSSLs that were reported previously, few were developed by using the male sterile line and were subjected to high-throughput genotyping by whole-genome resequencing to obtain accurate physical maps. It will be necessary to develop novel CSSLs with high-resolution physical maps.

Previously, our lab dissected yield-associated loci using an RIL derived from a cross between PA64s and 9311 (Gao et al. 2013). Since then, to facilitate QTL mapping and cloning, 3 sets of CSSLs were developed and were derived from three representative rice varieties: the first genome-sequenced *japonica* cultivar Nipponbare, an elite restore *indica* cultivar 9311, and a male sterile line PA64s with a mixed genetic background of *indica* and *japonica*. One set of CSSLs was developed from a cross between PA64s as the donor and Nipponbare as the recipient. The variety 9311 was used as recipient parent for the other two sets of CSSLs with Nipponbare and PA64s as donor parents, respectively. To obtain precise physical maps for QTL detection, the three sets of CSSLs were genotyped by high-throughput whole-genome resequencing. QTLs analysis for 11 major agronomic traits of the 3 sets of CSSLs was performed using the bin maps converted from the precise physical maps.

## Results

### Development of the CSSLs

A total of 311 polymorphic molecular markers were used to assist selection during CSSLs development (Additional file 1). Among them, 136 markers denoted as A, AB or AC were used for MAS in the population derived from PA64s × 9311 crosses, and 162 markers denoted as B, AB or BC were used for CSSL analysis on the population derived from a cross between 9311 and Nipponbare. Then, 138 markers denoted as C, AC or BC were used for the population derived from PA64s × Nipponbare crosses.

**Fig. 1:**
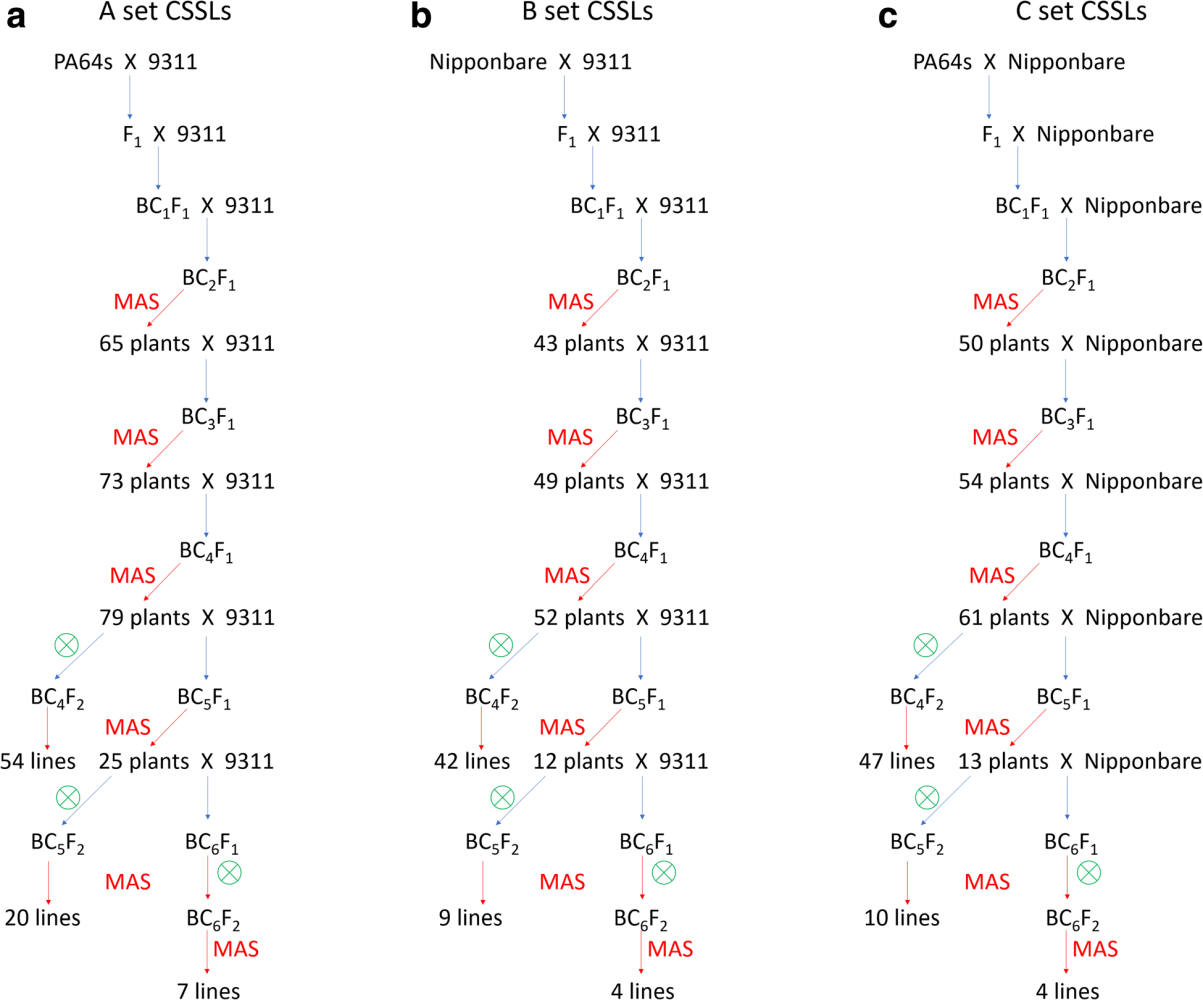
Flowchart of the procession of developing 3 sets of CSSLs. **a** Precession of developing A set CSSLs. **b** Precession of developing B set CSSLs. **c** Precession of developing C set CSSLs. The female parent is written in the front and the male parent in the back in hybrid combinations. The red arrow indicates that the genotypes of plants were identified by MAS. Numbers under the red arrow represent the selected individuals of lines by MAS. The green circle represents a self-cross

The development procedure for the 3 sets of CSSLs is shown in Fig. 1. Hybrid F_1_ plants, namely, LYP9, were generated from a cross between maternal PA64s and paternal 9311. Then, F_1_ plants were backcrossed twice with 9311 to produce BC_2_F_1_ generation. The first MAS was performed for BC_2_F_1_ generation with 136 polymorphic markers distributed on all 12 chromosomes, and 65 plants were selected from 93 individuals, in which a few of heterozygous substituted segments came from PA64s, while the major genomic regions were homozygous for 9311 alleles. Subsequently, the selected 65 plants from BC_2_F_1_ generation were backcrossed twice again with 9311 to generate BC_4_F_1_. Twelve individuals from each BC_3_F_1_ and BC_4_F_1_ line were genotyped using the markers on the target-substituted regions. In total, 54 plants from BC_4_F_1_ that had one to three heterozygous substituted segments were self-crossed to produce BC_4_F_2_. Then, 12 individuals from each BC_4_F_2_ were genotyped, and the homozygous substituted individuals on target regions were self-crossed to obtain 54 CSSLs. The remaining BC_4_F_1_ plants that had a few substituted segments that were continuously backcrossed with 9311 to generate BC_5_F_1_ and BC_6_F_1_. Then, 20 BC_5_F_2_ plants and 7 BC_6_F_2_ plants that were selected using MAS were self-crossed to obtain 27 CSSLs. Finally, a total of 81 CSSLs with a few substituted segments were developed, which were named A set CSSLs for easy description (Fig. 1a). Similarly, the other two sets of CSSLs, including 55 lines and 61 lines, were obtained with the same technique using 162 and 138 polymorphic markers, respectively (Fig. 1b and c). Meanwhile, 55 CSSLs with major genomic regions from 9311 had a few substituted segments from Nipponbare, which were named B set CSSLs. Then, 64 CSSLs were named C set CSSLs and contained a few substituted segments from PA64s, in which the major genomic regions were homozygous for Nipponbare.

### Characteristics of the CSSLs

**Fig. 2:**
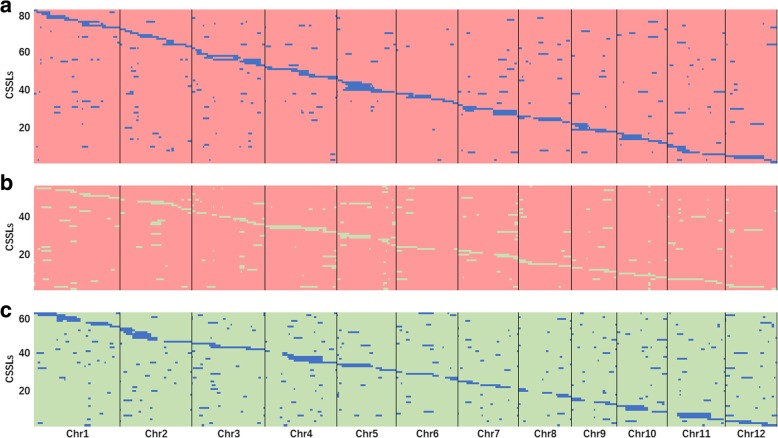
Graphic genotypes of 3 sets of CSSLs. **a** Genotypes of A set CSSLs. **b** Genotypes of B set CSSLs. **c** Genotypes of C set CSSLs. Different colors represent different genotypes: red, 9311; blue, PA64s; green, Nipponbare. Each row represents a CSSL. Chr is short for chromosome

**Fig. 3:**
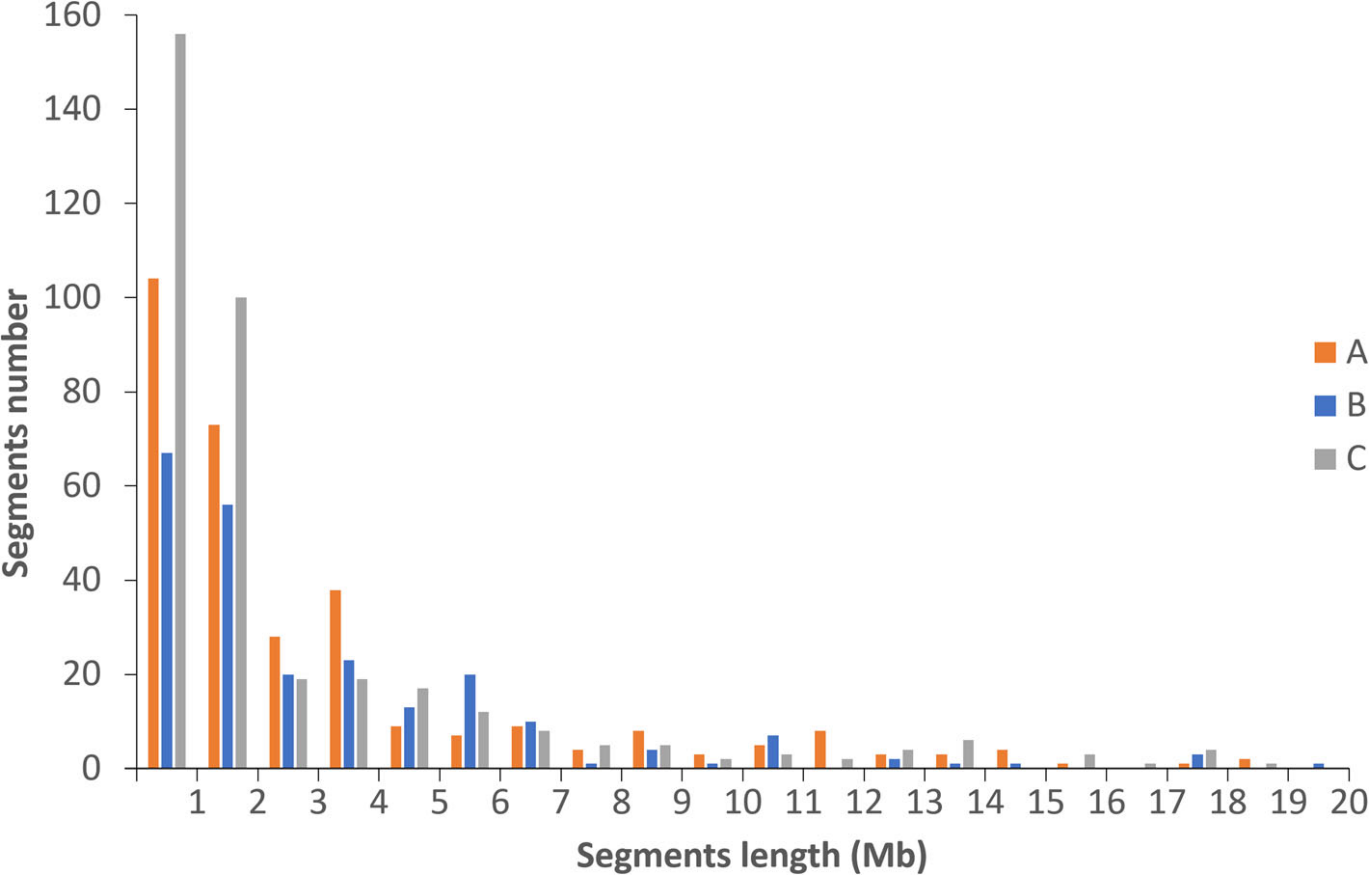
Distribution of the lengths of the substituted segments in the 3 sets of CSSLs. A, B and C represent A set CSSLs, B set CSSLs and C set CSSLs, respectively

To obtain accurate physical maps for the 3 sets of CSSLs, all of the CSSLs were subjected to high-throughput genotyping by whole-genome resequencing. Physical maps for the 3 sets of CSSLs were constructed using the physical locations of filtered SNPs (Fig. 2). The A set CSSLs containing 81 lines were found to carry 310 substituted segments, and there were at least 230 substituted segments in B set CSSLs and 367 substituted segments in C set CSSLs (Table 1). The average number of substituted segments was approximately 26, 19 and 31 per chromosome and ranged from 13 on chromosome 6 to 44 on chromosome 3, 10 on chromosome 6 to 30 on chromosome 3 and 5, and 24 on chromosome 5 to 38 on chromosome 4 for A, B and C sets of CSSLs, respectively. Statistical data for segment lengths showed that more than 61% segments were shorter than 2.00 Mb, while approximately 11% segments were longer than 7.00 Mb (Fig. 3). The total length of substituted segments was approximately 1152.61 Mb in A set CSSLs and was approximately 868.10 and 1177.96 Mb in B and C sets CSSLs, respectively (Table 1). The average length of substituted segments varied little, with an average of 3.72, 3.77 and 3.21 Mb in A, B and C sets of CSSLs, respectively. The total coverage length of substituted segments was 363.14 Mb in A set CSSLs, while that length was 325.70 and 331.40 Mb in B and C sets of CSSLs, respectively. The coverage rate of substituted segments was 97.3% in the A set CSSLs, which varied from 88.1% on chromosome 8 to 100.0% on chromosomes 2, 4, 5, 9 and 10. The coverage rate of substituted segments was nearly the same in B and C sets of CSSLs; it was 87.3% and 88.8%, respectively, and ranged from 67.3% to 100.0% on each chromosome.

**Table 1:** Distribution of substituted segments on 12 chromosomes for 3 sets of CSSLs

Chromosome	Number of segments			Segments length (Mb)			Average length (Mb)			Coverage length (Mb)			Coverage rate		
	A	B	C	A	B	C	A	B	C	A	B	C	A	B	C
1	38	29	37	129.81	123.45	117.72	3.42	4.26	3.18	42.00	39.45	40.72	97.1%	91.2%	94.1%
2	38	18	37	108.45	77.55	129.26	2.85	4.31	3.49	35.94	28.37	33.42	100.0%	78.9%	93.0%
3	44	30	37	131.20	81.61	109.86	2.98	2.72	2.97	36.16	32.64	36.41	99.3%	89.7%	100.0%
4	28	20	38	100.89	98.40	136.39	3.60	4.92	3.59	35.50	35.50	28.03	100.0%	100.0%	78.9%
5	24	30	24	111.55	95.62	84.14	4.65	3.19	3.51	29.96	24.86	27.41	100.0%	83.0%	91.5%
6	13	10	29	57.40	47.83	91.20	4.42	4.78	3.14	29.97	21.05	28.06	95.9%	67.3%	89.8%
7	25	23	30	108.86	84.12	75.46	4.35	3.66	2.52	27.83	23.50	26.60	93.8%	79.2%	89.6%
8	24	13	25	90.06	55.53	62.97	3.75	4.27	2.52	25.05	27.09	18.28	88.1%	95.2%	64.3%
9	15	14	30	72.87	56.25	71.59	4.86	4.02	2.39	23.01	21.09	17.90	100.0%	91.7%	77.8%
10	19	15	26	75.40	40.02	83.52	3.97	2.67	3.21	23.20	21.46	23.20	100.0%	92.5%	100.0%
11	26	17	26	84.54	57.41	114.19	3.25	3.38	4.39	27.13	27.13	23.34	93.5%	93.5%	80.4%
12	16	11	28	80.58	49.69	99.38	5.04	4.52	3.55	27.19	23.50	27.53	98.8%	85.4%	100.0%
Total	310	230	367	1152.61	868.10	1177.96	3.72	3.77	3.21	363.14	325.70	331.40	97.3%	87.3%	88.8%

A, B and C represent A set CSSLs, B set CSSLs and C set CSSLs, which derived from a cross between PA64s and 9311, Nipponbare and 9311, and PA64s and Nipponbare, respectively

### Phenotypic Variation

The phenotypic variations of 11 traits were observed between the 3 sets of CSSLs and their parents for QTL analysis. The t-test revealed that the differences between PA64s and Nipponbare were extremely significant concerning the panicle number (PN), primary panicle branch number (PPB), secondary panicle branch number (SPB), spikelet number per panicle (SN), grain length (GL), grain width (GW) and 1000-grain weight (TGW) in Shenzhen, as well as all traits but plant height (PH) and panicle length (PL) in Hangzhou. Both 9311 and Nipponbare showed significant differences in all traits but the flag leaf length (FLL) in Hangzhou and Shenzhen (Additional file 2: Table S1). The correlation of trait variation is shown in Additional file 2: Tables S2 to S7. The results show that extremely significant positive correlations were detected between the grain number and the secondary panicle branch number with the highest correlation coefficient, 0.8883 on average. In both Hangzhou and Shenzhen, 1000-grain weight showed an extremely significant positive correlation with grain length and grain width in the 3 sets of CSSLs, whereas it was negatively correlated with the spikelet number per panicle. Extremely significant positive correlations were also detected between plant height and panicle length in the 3 sets of CSSLs, except the B set CSSLs in Shenzhen. The phenotypic values of the 11 traits in the 3 sets of CSSLs were all found to be continuous and exhibited normal or skewed distribution patterns across both environments, indicating quantitative inheritance of the 11 characters and satisfying the demands for QTL analysis (Additional file 3: Figure S1).

### QTL Analysis for 11 Traits Based on CSSLs

**Fig. 4:**
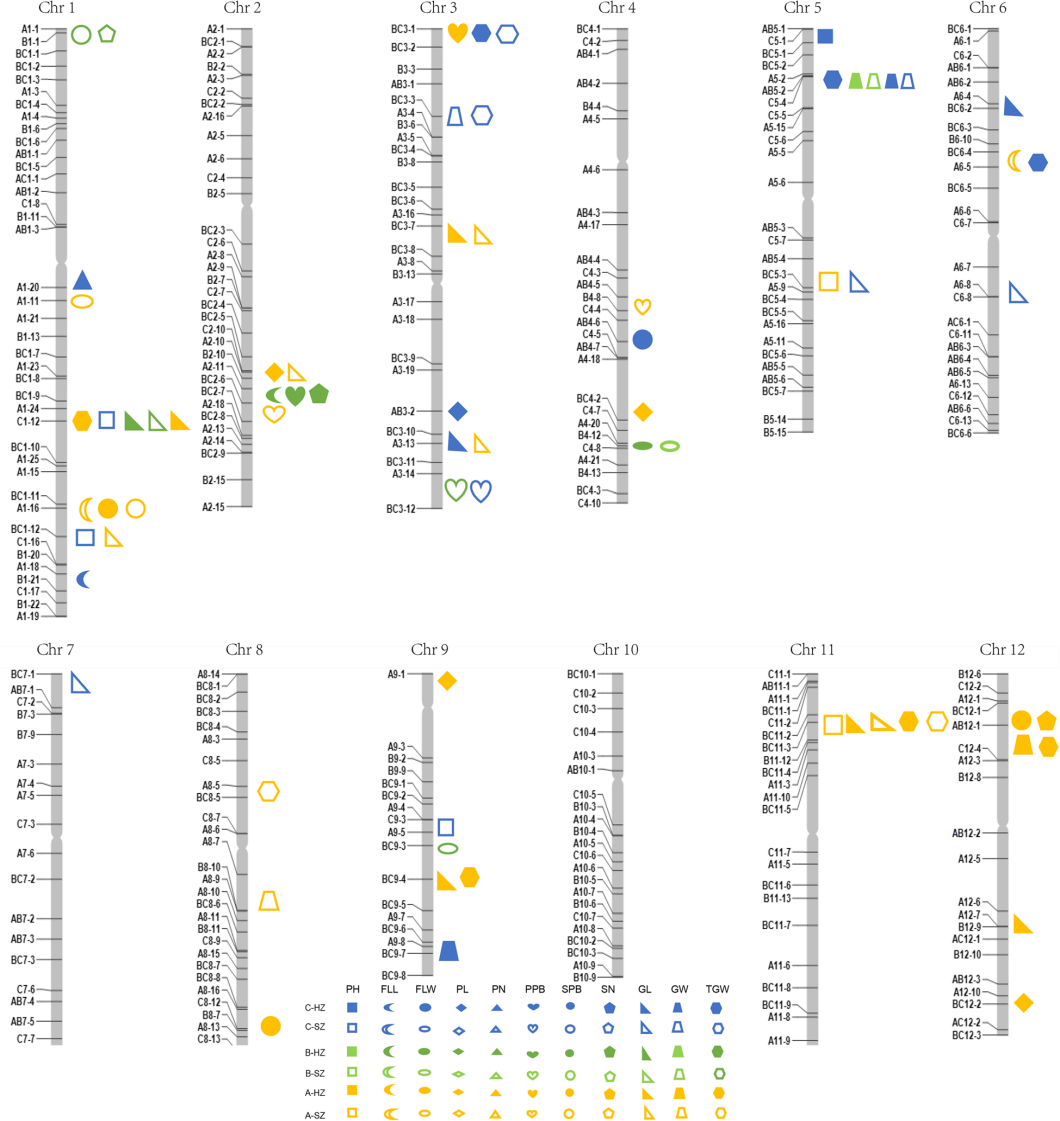
The positions of QTLs located on each chromosome. Markers for CSSLs development are shown in left. HZ and SZ represent Hangzhou and Shenzhen, respectively

To conduct the QTL analysis, resequencing-based physical maps of the 3 sets of CSSLs were converted into bin maps. A total of 71 QTLs for 11 traits in 3 sets of CSSLs were detected in both Hangzhou and Shenzhen and were distributed on 11 chromosomes, except for chromosome 10 (Fig. 4 and Table 2). Among the 34 QTLs that were detected in Shenzhen and the 37 QTLs in Hangzhou, 35 QTLs were detected in A set CSSLs under both environments, as well as 13 and 23 QTLs in the B and C sets of CSSLs, respectively. Eight QTLs were commonly detected in both Shenzhen and Hangzhou for the same phenotype, and 4 QTLs had overlapping interval locations in different populations. There were also 8 QTL clusters that were detected, including FLL and PPB, SPB and SN, GL and TGW, and GW and TGW, which coincided with significant correlations (Table 2, Additional file 2: Tables S2 to S7).

**Table 2:** QTLs for 11 traits detected in 3 sets of CSSLs in Shenzhen (SZ) and Hangzhou (HZ)

Trait	Site	Populations	QTL	Chromosome	Location (Mb)	LOD	PVE (%)^a^	Add^b^	Known QTL/ gene
PH	SZ	C	*qPH1–1*	chr1	30.0–30.5	5.0	22.8	−5.32	
PH	SZ	C	*qPH1–2*	chr1	38.0–38.4	6.3	26.3	5.88	*sd1*
PH	HZ	C	*qPH5–1*	chr5	0.8–1.3	3.7	19.1	7.53	
PH	SZ	A	*qPH5–2*	chr5	18.6–19.1	3.8	18.0	−4.42	
PH	SZ	C	*qPH9*	chr9	12.2–13.7	2.9	10.7	−2.93	
PH	SZ	A	*qPH11*	chr11	3.0–4.1	2.5	11.4	−4.88	
FLL	SZ	A	*qFLL1–1*	chr1	34.9–35.6	3.5	15.9	1.75	
FLL	HZ	C	*qFLL1–2*	chr1	42.0–43.2	2.5	16.8	−6.72	*qfll1*(Yan et al. 1999)
FLL	HZ	B	*qFLL2*	chr2	27.9–29.3	3.4	24.8	8.75	*qFLL5*(Wang et al. 2011)
FLL	SZ	A	*qFLL6*	chr6	9.6–10.5	3.3	14.8	3.05	
FLW	SZ	A	*qFLW1*	chr1	19.4–20.4	3.4	17.9	−0.11	
FLW	HZ	B	*qFLW4*	chr4	31.0–31.7	3.3	18.7	−0.21	*LSCHL4*
FLW	SZ	B	*qFLW4*	chr4	31.0–31.7	4.4	19.2	−0.25	*LSCHL4*
FLW	SZ	B	*qFLW9*	chr9	14.5–14.6	3.3	24.3	0.36	
PL	HZ	A	*qPL2*	chr2	26.5–26.5	4.3	12.7	−0.77	
PL	HZ	C	*qPL3*	chr3	30.0–30.4	4.4	27.5	1.20	*qPL-3*(Shen et al. 2009)
PL	HZ	A	*qPL4*	chr4	28.3–28.7	7.5	24.4	−0.72	*qPL-4-2*(Guo and Hong 2010)
PL	HZ	A	*qPL9*	chr9	0.0–5.7	4.4	13.2	0.60	
PL	HZ	A	*qPL12*	chr12	25.8–26.2	3.2	9.2	−1.02	
PN	HZ	C	*qPN1*	chr1	18.8–19.2	3.1	20.2	0.75	
PPB	HZ	B	*qPPB2–1*	chr2	27.9–29.3	7.1	44.8	2.67	
PPB	SZ	A	*qPPB2–2*	chr2	28.4–28.7	3.0	13.5	0.56	*qPPB3*(Gao et al. 2013)
PPB	HZ	A	*qPPB3–1*	chr3	0.9–1.4	3.4	17.2	0.56	
PPB	SZ	C	*qPPB3–2*	chr3	35.0–36.4	3.3	20.7	−0.75	
PPB	SZ	B	*qPPB3–3*	chr3	33.4–35.6	3.4	24.5	−0.88	
PPB	SZ	A	*qPPB4*	chr4	19.6–20.7	2.6	11.6	0.52	
SPB	SZ	B	*qSPB1–1*	chr1	0.0–0.5	4.0	28.7	−7.95	
SPB	HZ	A	*qSPB1–2*	chr1	35.1–36.2	3.5	15.4	5.64	*LAX1*
SPB	SZ	A	*qSPB1–2*	chr1	35.1–36.2	3.7	16.3	5.90	*LAX1*
SPB	HZ	C	*qSPB4*	chr4	22.8–23.4	4.0	30.5	3.57	
SPB	HZ	A	*qSPB8*	chr8	26.2–27.9	3.0	13.4	3.01	
SPB	HZ	A	*qSPB12*	chr12	5.7–6.9	2.6	11.0	2.09	
SN	SZ	B	*qSN1*	chr1	0.0–0.5	2.8	21.0	−25.21	
SN	HZ	B	*qSN2*	chr2	27.9–29.3	2.6	19.8	47.00	*qGW-2b* (Wang et al. 2011)
SN	HZ	A	*qSN12*	chr12	5.7–6.9	2.7	14.1	6.61	
GL	HZ	A	*qGL1–1*	chr1	28.5–30.0	3.6	27.3	0.30	
GL	SZ	B	*qGL1–2*	chr1	28.1–30.0	3.5	20.1	0.26	
GL	HZ	B	*qGL1–2*	chr1	28.1–30.0	3.3	19.6	0.22	
GL	SZ	A	*qGL1–3*	chr1	37.7–38.0	3.6	10.4	0.21	
GL	SZ	A	*qGL2*	chr2	26.5–27.4	3.3	9.3	−0.28	
GL	HZ	A	*qGL3–1*	chr3	15.9–18.2	3.5	13.6	0.14	
GL	SZ	A	*qGL3–1*	chr3	15.9–18.2	4.0	16.3	0.26	
GL	HZ	C	*qGL3–2*	chr3	30.0–31.6	3.5	17.7	0.16	
GL	SZ	A	*qGL3–3*	chr3	31.1–32.5	4.3	17.8	−0.32	
GL	SZ	C	*qGL5*	chr5	18.4–18.9	4.8	17.5	0.31	
GL	HZ	C	*qGL6–1*	chr6	6.6–7.1	3.4	17.0	0.16	
GL	SZ	C	*qGL6–2*	chr6	21.1–22.1	4.5	7.6	0.13	*qGL6.2*(Bian et al. 2010)
GL	SZ	C	*qGL7*	chr7	0.5–2.1	4.9	8.3	0.16	*qGL7*(Bian et al. 2010)
GL	HZ	A	*qGL9*	chr9	15.4–16.1	3.3	8.8	−0.21	*qGL9.1*(Bian et al. 2010)
GL	SZ	A	*qGL11*	chr11	2.9–5.8	3.1	8.7	−0.21	
GL	HZ	A	*qGL11*	chr11	2.9–5.8	4.1	29.2	−0.26	
GL	HZ	A	*qGL12*	chr12	19.8–20.6	5.2	14.8	−0.30	
GW	SZ	C	*qGW3*	chr3	7.9–8.6	3.0	11.5	0.06	*qGW3*(Wang et al. 2011)
GW	HZ	B	*qGW5*	chr5	4.2–5.5	3.5	25.4	−0.12	*GW5*
GW	SZ	B	*qGW5*	chr5	4.2–5.5	4.8	33.3	−0.14	*GW5*
GW	HZ	C	*qGW5*	chr5	5.3–5.5	4.8	44.8	0.14	*GW5*
GW	SZ	C	*qGW5*	chr5	5.3–5.5	6.3	27.6	0.11	*GW5*
GW	SZ	A	*qGW8*	chr8	17.0–17.7	4.3	21.1	−0.06	
GW	HZ	C	*qGW9*	chr9	21.4–21.9	2.6	15.7	−0.13	
GW	HZ	A	*qGW12*	chr12	4.5–5.5	3.0	12.1	−0.04	
TGW	HZ	A	*qTGW1*	chr1	28.5–30.0	4.0	12.0	1.40	
TGW	HZ	C	*qTGW3–1*	chr3	0.0–1.12	3.6	14.4	−0.98	
TGW	SZ	C	*qTGW3–1*	chr3	0.0–1.12	4.6	23.7	−1.34	
TGW	SZ	C	*qTGW3–2*	chr3	7.9–8.6	3.2	15.8	1.06	
TGW	HZ	C	*qTGW5*	chr5	5.3–5.5	4.9	20.9	1.37	*GW5*
TGW	HZ	C	*qTGW6*	chr6	11.7–19.4	6.3	28.5	1.46	
TGW	SZ	A	*qTGW8*	chr8	8.6–8.7	4.4	19.2	−1.21	
TGW	HZ	A	*qTGW9*	chr9	15.4–16.1	6.7	22.0	−2.35	
TGW	SZ	A	*qTGW11*	chr11	2.9–5.8	3.8	16.0	−1.73	*qTGW11.1*(Bian et al. 2010)
TGW	HZ	A	*qTGW11*	chr11	2.9–5.8	5.5	17.3	−1.41	*qTGW11.1*
TGW	HZ	A	*qTGW12*	chr12	5.7–6.9	3.2	9.3	−1.07	

^a^The percentage of phenotypic variation explained by the detected QTL

^b^Additive effects; The positive value indicates that alleles from recurrent parent increase the effect

Compared with the previously identified QTLs by physical location, 22 of the 71 QTLs were reported in the previous studies, and the remaining 49 QTLs were unique. Among the 22 reported QTLs, the interval regions of the four QTLs overlapped with the locations of four known genes (Additional file 4: Figure S2). For the QTL with the largest effect on plant height in C set CSSLs, *qPH1–2* can explain 26.3% of the phenotypical variation and was located in the region from 38.0 to 38.5 Mb on chromosome 1, which contained *sd1*, the rice green revolution gene that is responsible for rice semi-dwarfness (Sasaki et al. 2002). In the B set CSSLs, the Nipponbare alleles increased the flag leaf width at *qFLW4* under two environments, which overlapped with the location of *Nal1/ LSCHL4* that controlled leaf width and yield (Fujita et al. 2013; Qi et al. 2008; Zhang et al. 2014). For the secondary panicle branch number, *qSPB1–2* was mapped to the region that ranged from 35.1 to 36.2 Mb on chromosome 1, while *LAX1*, a gene controlling panicle branch development, was previously cloned (Komatsu et al. 2003). Finally, the major QTL *qGW5* with Nipponbare alleles that increased the grain width was detected in B and C set of CSSLs under two environments, while *GW5/GSE5* was located in the region by previous studies (Duan et al. 2017; Liu et al. 2017).

### Validation and Fine-Mapping of *qGL1*

**Fig. 5:**
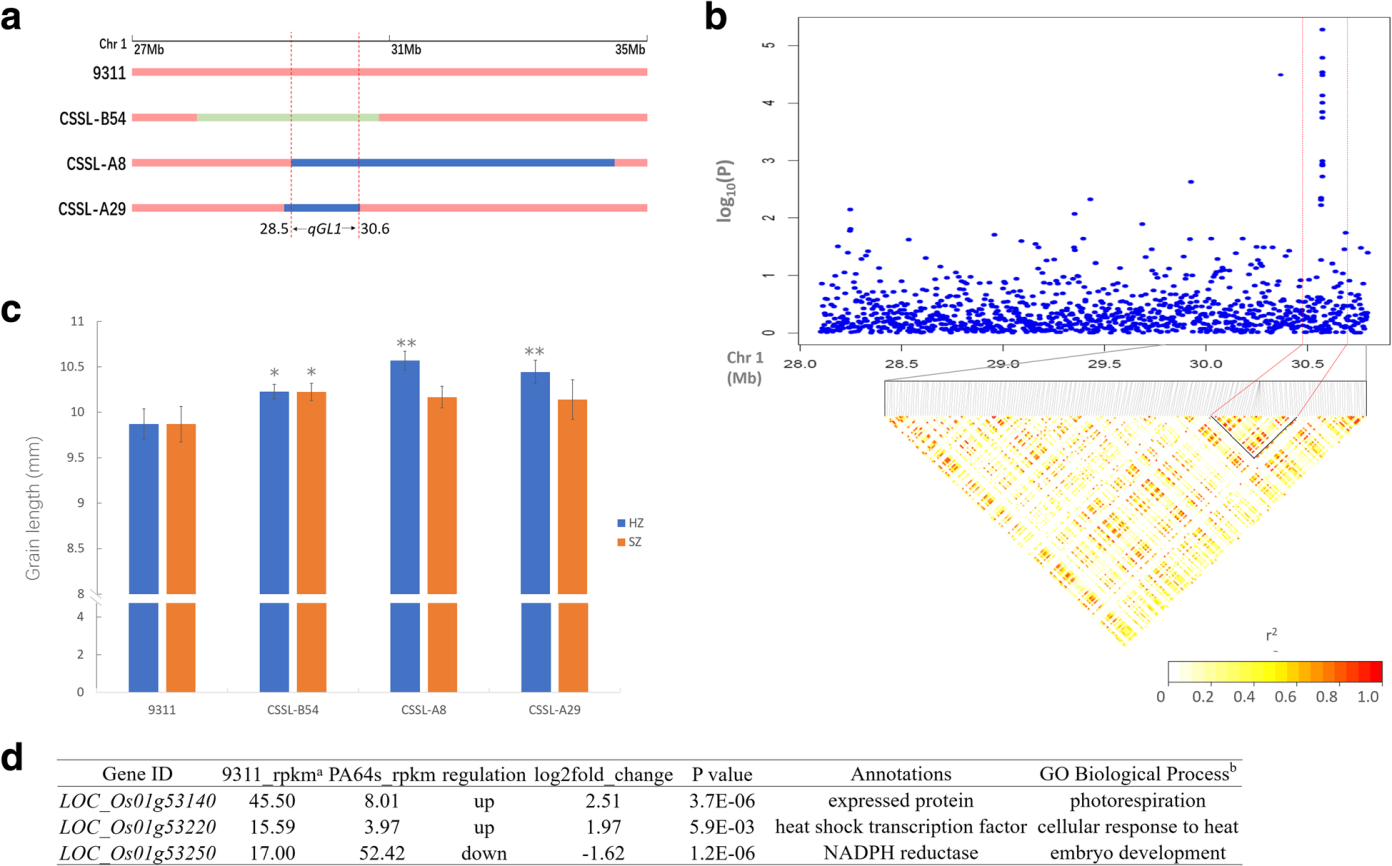
Fine mapping of *qGL1*. **a** the interval of *qGL1* detected in A set CSSLs and B set CSSLs. Different colors represent different genotypes: red, 9311; blue, PA64s; green, Nipponbare. Dashed lines indicate the overlap regions in different CSSLs. **b** Local Manhattan plot (top) and LD heatmap (bottom) surrounding the peak on chromosome 1. Dashed lines indicate the candidate region for the peak. **c** variation of grain length between 9311 and 3 CSSLs. HZ and SZ represent Hangzhou and Shenzhen, respectively. Error bars are s.d. * and ** indicate the least significant difference at 0.05 and 0.01 probability level compared with the recurrent parent in SZ or HZ, respectively. **d** Differential gene expression and annotation. ^a^rpkm is short for Reads Per Kilobase per Million mapped reads; ^b^information refers to its Arabidopsis homologs at The Arabidopsis Information Resource (TAIR)

*qGL1* had a stable effect in B set CSSLs under both environment and was also detected in A set CSSLs in Hangzhou (Fig. 4 and Table 2). As CSSL-A8, CSSL-A29 and CSSL-B54 had overlapped regions on *qGL1* location, the interval of *qGL1* could be narrowed to the region of 2.1 Mb which ranged from 28.503 Mb to 30.611 Mb on chromosome 1 (Fig. 5a). To identify genes associated with grain length and further reduce the interval of *qGL1*, we performed GWAS by analyzing a natural rice population, consisting of 317 rice varieties that were collected from the 3000 Rice Genomes Project (Wang et al. 2018). Using the efficient Mixed-Model, 8 SNPs at *qGL1* location on chromosome 1 were identified to be significantly associated with grain length. Then, we used pairwise linkage disequilibrium (LD) correlations to estimate a candidate region from 30.492 Mb to 30.652 Mb for *qGL1* (Fig. 5b). Hence, both our GWAS and CSSLs results confirmed that the interval of *qGL1* could be narrowed to a 119 kb region ranged from 30.492 Mb to 30.611 Mb.

There were 18 annotated genes within this 119 kb interval based on Rice Genome Annotation Project Website (http://rice.plantbiology. msu.edu/) (Additional file 2: Table S8). Among the 18 genes, 3 genes expressed different significantly between 9311 and PA64s in young panicle tissues based on RNA-seq data. The expression of *LOC_Os01g53140* and *LOC_Os01g53220* in 9311 were higher than that in PA64s, while the expression of *LOC_Os01g53250* was significantly higher in PA64s (Fig. 5d). Moreover, sequence variations of those genes between 9311 and Nipponbare were identified and expressions at RNA level were also analyzed in young panicles between 9311 and CSSL-B54 (Additional file 6: Figure S4). Both 9311 and Nipponbare had amino acid variations of these three candidate genes, which were caused by SNPs on exons. Nipponbare and CSSL-B54 showed lower expression level on *LOC_Os01g53140* and *LOC_Os01g53220* compared with 9311, which was coincided with the RNA-seq data. Similarly, the expression of *LOC_Os01g53250* was higher in Nipponbare and CSSL-B54 than that in 9311. *LOC_Os01g53220* has previously been designated as *OsHsfC1b, which* is a heat shock transcription factor and plays a role in ABA-mediated salt stress tolerance in rice (Mittal et al. 2009; Schmidt et al. 2012). *LOC_Os01g53250* product is a NADPH reductase whose homologous gene in *Arabidopsis* is *ATR3*. ATR3 is NADPH-dependent diflavin oxidoreductase 1, which is involved in embryo development ending in seed dormancy and oxidation-reduction process (Varadarajan et al. 2010). The product of *LOC_Os01g53140* is an expressed protein. Its homologous protein in *Arabidopsis* is a neuronal acetylcholine receptor subunit alpha-5 which is subunit of mitochondrial respiratory chain complex I and is involved in photorespiration (Jennifer et al. 2010). Collectively, on the basis of our results, we suggest that *qGL1* is located in a 119 kb region on chromosome 1 and *LOC_Os01g53140* and *LOC_Os01g53250* are the two most likely candidate genes for *qGL1.*

## Discussion

CSSLs are the ideal population for detecting the precise QTLs that control complex traits by eliminating genetic background noise (Nadeau et al. 2000; Qiao et al. 2016; Tian et al. 2006; Ye et al. 2010; Zhou et al. 2017). On the other hand, because CSSLs contain a few substituted chromosome segments from donor parent, they can be used as NILs themselves or used to develop NILs with additional cross with the recurrent parent, which are fast and powerful tools for QTL validation and isolation (Ebitani et al. 2005; Mulsanti et al. 2018; Zhu et al. 2017). Under a certain condition, CSSLs are limited for QTL detection. Because serval CSSLs carry some long-substituted chromosome segments that can range between 10 and 20 Mb, they can hardly detect the QTLs that are close to each other with opposite effects. Here, we developed 3 sets of CSSLs using repeated elite parents, which may be much easier for QTL detection and mutual authentication.

Because there are different genetic backgrounds among lines or individuals, the epistatic effects between QTLs for many quantitative traits estimated by using conventional experimental materials are confounded with other genetic factors (Zhu et al. 2015a). Due to the single segment substitution, CSSLs may be ideal materials for verifying and researching the interaction between two QTLs in the additive effect and the epistatic effect by a cross between two related CSSLs. In rice, a number of QTLs and their epistasis on traits, such as plant height, heading date, and yield components, have been well analyzed by using CSSLs or secondary F_2_ populations that were derived from CSSLs (Chen et al. 2014; Kubo et al. 2008; Wang et al. 2017; Yang et al. 2018; Zhu et al. 2015a). On the other hand, CSSLs can also be used for QTL pyramiding in plant breeding. *Gn1a* can increase the grain number and improve rice yield simultaneously through inhibiting *OsCKX2* gene expression in the inflorescence meristem, while the green revolution gene *sd1* reduces bioactive gibberellin (GA) abundance, which results in semi-dwarfism to resistant to lodging. NIL-*sd1* + *Gn1a* generated by crossing NIL-*Gn1a* and NIL-*sd1* obtains an increased grain number but decreased plant height compared with Koshihikari, which provides a new strategy for improving crop yield through QTL pyramiding (Ashikari et al. 2005). The rational design rice which pyramided the good appearance genes *GS3* and *qSW5* from 9311 and the superior quality genes *Wx* and *ALK* from Nipponbare showed significantly improved grain appearance and quality compared with Teqing (Zeng et al. 2017). *GW5/GSE5* is a major QTL that regulates grain width and weight predominantly by influencing cell proliferation in spikelet hulls (Duan et al. 2017; Liu et al. 2017). *NAL1/LSCHL4*, which regulates the development of the vascular bundle conferred leaf width, enhances the grain productivity of *indica* cultivars by increasing the number of secondary panicle branches and the spikelet number per panicle with declines in the seed-setting rate and in the 1000-grain weight (Fujita et al. 2013; Qi et al. 2008; Zhang et al. 2014). In this study, the confidence intervals of *qFLW4* and *qGW5* that were detected in the B set CSSLs overlapped with the locations of *Nal1* and *GW5*, respectively (Table 2). The grain width performance of CSSL-B35 and CSSL-B36, which carried Nipponbare alleles segments at *qGW5*, showed significant differences compared with 9311 in across two environments, as well as the flag leaf width of CSSL-B10 at *qFLW4* (Additional file 4: Figure S2 D and E). The increasing effects of Nipponbare alleles at both sites suggested a possibility of improving the 9311 yield by pyramiding the good appearance QTL *qFLW4* and *qGW5* from a cross between CSSL-B10 and CSSL-B35 or CSSL-B36.

PCR-based molecular markers are useful tools for selection during CSSLs development. However, they can hardly detect the small substituted segments or determine the length of substituted segments because the number of polymorphic markers used for selection is limited. Resequencing-based high-throughput genotyping technology can overcome these shortcomings by improving the resolution of the physical map accurately and reducing laborious and time-consuming work compared with the conventional molecular marker-based genotyping method (Watanabe et al. 2018; Xu et al. 2016; Zhang et al. 2011; Zhu et al. 2015b). As illustrated in Additional file 5: Figure S3, double-crossovers in CSSL-A35, CSSL-A44 and other CSSLs were detected based on throughput resequencing technology, while these double-crossovers can hardly detect by PCR-based molecular markers for the limited number of molecular markers. In this study, we developed 3 sets of CSSLs by molecular markers for selection and constructed high-density physical maps of the 3 sets of CSSLs by the whole-genome resequencing approach for high-throughput genotyping. A total of 197 lines were selected by 311 molecular markers for the development of the 3 sets of CSSLs. The coverage rates of substituted segments were 97.3%, 87.3% and 88.8% in the A, B and C sets of CSSLs respectively (Table 1). The number and length of substituted segments in 3 sets of CSSLs were also counted precisely using high-throughput genotyping data (Fig. 3), which provided a platform for genetic research and molecular rice breeding.

Due to significant phenotypic variations and the strong potential for heterosis between *indica* and *japonica* subspecies, many populations included some CSSLs that were derived from crosses between *indica* variety and *japonica* variety have been constructed for QTL mapping and gene cloning (Song et al. 2007; Wei et al. 2010; Zhou et al. 2016). To facilitate QTL detection and isolation, we developed 3 sets of CSSLs from three types of rice varieties: a typical *japonica* cultivar Nipponbare, an elite *indica* cultivar restore line 9311, and a middle type cultivar PA64s with a mixed genetic background of *indica* and *japonica*. To evaluate the potential advantages of the 3 sets of CSSLs for QTL detection, the phenotypic variations of 11 agronomic traits were observed between the 3 sets of CSSLs and their parents in Hangzhou and Shenzhen. Among the 71 QTLs detected in the 3 sets of CSSLs for 11 agronomic traits, 22 QTLs, including 4 known genes, were located in the vicinity of QTLs that affect these traits detected in the other populations, which suggested less epistatic effects of these QTLs in varied genetic backgrounds (Fig. 4 and Table 2). Because of the pleiotropic effects or the tight linkage of genes, multiple QTLs affecting related traits were mapped to the same region to form QTL clusters, such as *qSPB12*, *qTGW12* and *qSN12* on chromosome 12 in A set CSSLs and *qFLL2*, *qPPB2–1* and *qSN2* on chromosome 2 in B set CSSLs, which also coincided with significant correlations, thus indicating that the relationship among these quantitative traits was intricate.

Although CSSLs are powerful tools for QTL detection, the intervals of QTLs are often too large, which are determined by the length of substituted segments. GWAS based on large-scale resequencing provide a powerful platform for finding genetic variants that can be directly used for crop improvement (Yano et al. 2016). In this study, the interval of *qGL1* was located to the region of 2.1 Mb by two CSSLs. In parallel, we also found 8 associated SNPs at *qGL1* location through GWAS and estimated a candidate region from 30.492 Mb to 30.652 Mb for *qGL1* by LD correlations. As the confidence interval resulted from GWAS is overlapped with the CSSLs location, the interval of *qGL1* was narrowed to the region of 119 kb which ranged from 30.492 Mb to 30.611 Mb on chromosome 1. The expression profiles of the diverse varieties of the collection in a particular tissue can more accurately aid in the determination of candidate genes, and annotation of candidate gene in the local region can also provide evidences for gene function (Xuehui et al. 2012). In this study, 3 genes expressed different significantly in the candidate region in young panicle tissue between 9311 and CSSL-B54. Among the 3 differentially expressed genes, *LOC_Os01g53140* and *LOC_Os01g53250* are the two most likely candidate genes for *qGL1*, as they may be involved in embryo development and energy metabolism, respectively, according to homologous alignment and gene function annotation.

## Conclusions

In this study, three sets of CSSLs were developed containing 81, 55 and 64 lines, respectively, which provided a platform for basic genetic research and offered ideal materials for molecular rice breeding. High-throughput whole-genome resequencing strategy made the physical maps for the 3 sets of CSSLs more accurate for precise information on location and length of substituted segments, which facilitated novel QTLs discovery. Combined with GWAS on grain length and expression profiles on young panicle tissues, *qGL1* detected in CSSLs was fine mapped within a 119 kb region on chromosome 1 offering a new gene resource for improving grain shape.

## Methods

### Plant Materials

Nipponbare was the first map-based sequenced *japonica* cultivar. Restorer line 9311and photothermo-sensitive male sterile line PA64s are the parents of pioneer super hybrid rice LYP9, which has been widely cultivated for commercial production in China. MAS was used for the development of three sets of CSSLs during crossing and backcrossing. Three parents and the populations derived from the three parents were grown annually at the experimental farm of China National Rice Research Institute in Hangzhou (119°54′ E, 30°04′ N) and Lingshui (110°00′ E, 18°31′ N).

### DNA Extraction and Molecular Analysis

CTAB method was used for the extraction of genomic DNA from fresh young leaves of each individual (Rogers and Bendich 1988). The DNA amplification was performed by PCR (Polymerase Chain Reaction) with the protocol as follows: predenaturation at 94 °C for 4 min; 38 cycles of denaturation at 94 °C for 30 s, annealing at 55–60 °C for 30 s and extension at 72 °C for 30 s, with a final extension at 72 °C for 5 min. The reactions were carried out in 96-well PCR plates in 10 μl volumes containing 50–100 ng template DNA, 0.2 μmol/L of each primer and 5 μl of 2× Taq Master Mix (Vazyme Inc.). Electrophoresis of the amplification products were carried out on 4% agarose gels and photographed using GelDoc XR Gel Documentation System (Bio-Rad Inc.).

Most polymorphic molecular markers used for MAS were SSR markers that were selected from the Gramene Markers Database (McCouch et al. 2002; Project IRGS 2005). The other markers used were InDels markers developed from a BLASTN alignment between the genome sequence of three parents. Primers were designed on Primer3 web (http://primer3.ut.ee/).

### High-Throughput Genotyping by Whole-Genome Resequencing

At least 1 μg of genomic DNA from each sample of CSSLs was used for resequencing with HiSeq PE150 strategy on Illumina HiSeq Xten. All DNA samples had backups for verification. Approximately 10× coverage sequence reads were generated for each CSSL. The *O. sativa* cv. Nipponbare IRGSP 1.0 reference genome was used for alignment (Kawahara et al. 2013). SNP calling was carried out with SAMtools (Li et al. 2009). A hidden Markov model was used to impute genotypes of recombinational chromosome fragments based on the observed genotypes of SNPs, following the previous method (Xie et al. 2010). The recombinational fragments less than 300 kb were identified as missing data in our study, and a crossover was defined between two adjacent blocks with different genotypes. An interval between two adjacent crossovers in the whole CSSL population was regarded as a bin (Zhou et al. 2015).

### Phenotypic Assessment for CSSLs

The 3 sets of CSSLs and their parents were grown for phenotypic assessment at the experimental farm in Hangzhou (119°54′ E, 30°04′ N) and Shenzhen (114°03′ E, 22°32′ N) in 2017. Twenty-five-day-old seedlings of each line were transplanted into an eight-row plot with six plants per row and spacing of 20 × 20 cm. The field management followed normal agricultural practice. The measurements of the plant height, panicle number, flag leaf length and flag leaf width were performed directly in the field at 25 days after heading. For all those traits, five plants of each line were sampled from the middle of each plot, and the main culm of each plant was chosen for trait measurement. At maturity, the main panicles of the individuals were harvested from 5 plants in the middle of each plot to measure the panicle and grain-related traits in the laboratory. For the five main panicles of each line, panicle length was measured with rulers; the primary panicle branch number, secondary panicle branch number and spikelet number per panicle were counted manually. Fully filled grains per panicle were used to calculate the grain length, grain width and 1000-grain weight using the WinSEEDLE Analysis System (Regent Instruments Inc.). Basic statistical analysis and correlation analysis were conducted with SAS 8.0 (SAS Institute). Five replications were performed for each trait.

### QTL Analysis for 11 Major Agronomic Traits Based on CSSLs

Bin maps were converted from the physical maps of 3 sets of CSSLs for QTL analysis. Most donor segments between or among the different lines of each CSSL have a little overlap. To perform QTL analysis easily and precisely, the overlapping chromosome segments of the CSSLs were utilized to delineate smaller segment sizes that were described as bins (Huang et al. 2009b). The QTL analysis was performed from these bins that served as genetic markers using QTL IciMapping V4.0 (www.isbreeding.net/software/). The likelihood ratio test based on stepwise regression for the additive QTL (RSTEP-LRT-ADD) method was employed for power analysis. The mapping parameters of probability in stepwise regression was set at 0.001, and the multicollinearity control by condition number was 1000 as the default. The LOD threshold for each dataset was set based on a permutation test (1000 permutations, *P* = 0.05). When the QTL LOD score was larger than 2.5, the QTL was designated having a major effect.

### Genome-Wide Association Study and Linkage Disequilibrium

A total of 317 varieties are selected from 3010 diverse accessions of Asian cultivated rice and the SNPs information was acquired in public database (Wang et al. 2018). GWAS for the grain length were carried out using Efficient Mixed-Model Association eXpedited (EMMAX) software package (Hyun Min et al. 2010). The LD between SNPs in the 317 varieties was evaluated using squared Pearson’s correlation coefficient (*r*^*2*^) as calculated with the -*r*^*2*^ command in the software PLINK. The LD heatmap surrounding peaks in the GWAS was constructed using LDheatmap R package (Shin et al. 2006).

### RNA-Seq Analysis

We extracted total RNA from the 2–5 cm long young panicle tissues of rice plants with an RNA extraction kit (Trizol reagent, Invitrogen). The libraries were sequenced on a HiSeq. PE150 paired-end reads were generated. The sequencing reads were aligned to the Nipponbare (*Oryza sativa* L. spp. *japonica*) reference genome (http://rice.plantbiology.msu.edu, TIGR Version 7). A gene was considered expressed if the reads per kilobase of transcript model per million mapped reads was > 0.4. Differential gene expression between sample groups was analyzed using Cufflinks software for each gene. Genes were considered differentially expressed if the |log2(fold change)| ≥1 and FDR (false discovery rate) < 0.05.

### Quantitative Real-Time RT-PCR Analysis

Total RNA was isolated from the 2 cm long young panicle tissues in Shenzhen with an RNA extraction kit (Trizol reagent, Invitrogen). cDNA was synthesized using a Rever Tra Ace® qPCR-RT kit (TOYOBA). Real time PCR amplification mixtures (10 μl) contained 50 ng template cDNA, 2 × SYBR Green PCR Master Mix (invitrogen), and 200 nM forward and reverse primers. Reactions were run on a StepOnePlus™ Real-Time PCR System (Thermo Fisher Scientific). The relative expression level of each transcript was obtained by comparing to the expression of the *OsActin1* gene. Primers for candidate genes and *OsActin1* are listed in Additional file 2: Table S9.

## Additional files

Additional file 1: Primers used for MAS during CSSLs development. (XLSX 35 kb)

Additional file 2: **Table S1.** Eleven traits of Nipponbare, PA64s and 9311 observed in Shengzhen (SZ) and Hangzhou (HZ). Mean ± SD. (*n* = 5). * and ** indicate the least significant difference at 0.05 and 0.01 probability level compared with Nipponbare in SZ or HZ, respectively. **Table S2.** Correlation analysis of 11 traits for A set CSSLs in Hangzhou (HZ). *P* values are shown below and bolds mean *P* < 0.01. **Table S3.** Correlation analysis of 11 traits for A set CSSLs in Shenzhen (SZ). P values are shown below and bolds mean *P* < 0.01. **Table S4.** Correlation analysis of 11 traits for B set CSSLs in Hangzhou (HZ). P values are shown below and bolds mean *P* < 0.01. **Table S5.** Correlation analysis of 11 traits for B set CSSLs in Shenzhen (SZ). P values are shown below and bolds mean *P* < 0.01. **Table S6.** Correlation analysis of 11 traits for C set CSSLs in Hangzhou (HZ). P values are shown below and bolds mean *P* < 0.01. **Table S7.** Correlation analysis of 11 traits for C set CSSLs in Shenzhen (SZ). P values are shown below and bolds mean *P* < 0.01. **Table S8.** Candidate genes in the 119 kb interval for *qGL1*. Bolds indicate differential expressed genes based on RNA-seq data. **Table S9.** Primers used for sequence identification. (DOCX 45 kb)

Additional file 3: **Figure S1.** Variation of the phenotypic traits in 3 sets of CSSLs. (TIF 1322 kb)

Additional file 4: **Figure S2.** Sequence difference and phenotypic variations on four reported genes. **A.** Difference sites between the related two parents. The difference sites are shown in red. Amino acids variations are listed in parenthesis. Empty blocks and blue blocks represent non-coding regions and exons, respectively. **B-E.** Phenotypic variation between CSSLs individuals and their recurrent parents. **B.** CSSL-A9 and CSSL-A53 harbor *Lax1* of PA64s allele at *qSPB1–2* locus. **C.** CSSL-C19 harbors *Sd1* of PA64s allele at *qPH1–2* locus. **D.** CSSL-B35 and CSSL-B36 harbor *GW5* of Nipponbare allele at *qGW5* locus. **E.** CSSL-B10 harbors *Nal1* of Nipponbare allele at *qFLW4* locus. Error bars are s.d. * and ** indicate the least significant difference at 0.05 and 0.01 probability level compared with the recurrent parent in SZ or HZ, respectively. NPB represents Nipponbare. (TIF 211 kb)

Additional file 5: **Figure S3.** Double-crossovers in CSSLs. Different colors represent different genotypes: red, 9311; blue, PA64s; green, Nipponbare. Double-crossovers were shown in black panes. (TIF 1447 kb)

Additional file 6: **Figure S4.** Quantitative real-time RT-PCR analysis and Sequence variations of predicted genes. **A-C.**RNA relative expression level of 9311, Nip and CSSL-B54 in young panicle for *LOC_Os01g53140*, *LOC_Os01g53140* and *LOC_Os01g53250*, respectively. **D-F.** Sequence variations between Nip and 9311 for *LOC_Os01g53140*, *LOC_Os01g53140* and *LOC_Os01g53250*, respectively. Values represent means ± SD of three independent assays. ** indicate the least significant difference at 0.01 probability level compared with 9311. NPB represents Nipponbare. SNPs on the exons are shown in bold and Amino acids variations are listed in parenthesis. (TIF 3099 kb)

## Abbreviations

CSSLs: Chromosome segment substitution lines
DH: Doubled haploid
FLL: Flag leaf length
FLW: Flag leaf width
GA: Gibberellin acid
GL: Grain length
GW: Grain width
GWAS: Genome-wide association study
Indels: Insertions and deletions
LD: Linkage disequilibrium
MAS: Marker-assisted selection
PCR: Polymerase chain reaction
PH: Plant height
PL: Panicle length
PN: Panicle number
PPB: Primary panicle branch number
QTLs: Quantitative trait loci
RILs: Recombinant inbred lines
SN: Spikelet number per panicle
SNPs: Single nucleotide polymorphisms
SPB: Secondary panicle branch number
SSR: Simple sequence repeat
TGW: 1000-grain weight
