# Supplementary material for: Development of Three Sets of High-Throughput Genotyped Rice Chromosome Segment Substitution Lines and QTL Mapping for Eleven Traits
Source: Rice (N Y). 2019 May 10;12:33. doi: 10.1186/s12284-019-0293-y (PMC6510774; doi:10.1186/s12284-019-0293-y)
Supplement: Supplementary file 2 — Table S1. Eleven traits of Nipponbare, PA64s and 9311 observed in Shengzhen (SZ) and Hangzhou (HZ). Mean ± SD. (n = 5). * and ** indicate the least significant difference at 0.05 and 0.01 probability level compared with Nipponbare in SZ or HZ, respectively. Table S2. Correlation analysis of 11 traits for A set CSSLs in Hangzhou (HZ). P values are shown below and bolds mean P < 0.01. Table S3. Correlation analysis of 11 traits for A set CSSLs in Shenzhen (SZ). P values are shown below and bolds mean P < 0.01. Table S4. Correlation analysis of 11 traits for B set CSSLs in Hangzhou (HZ). P values are shown below and bolds mean P < 0.01. Table S5. Correlation analysis of 11 traits for B set CSSLs in Shenzhen (SZ). P values are shown below and bolds mean P < 0.01. Table S6. Correlation analysis of 11 traits for C set CSSLs in Hangzhou (HZ). P values are shown below and bolds mean P < 0.01. Table S7. Correlation analysis of 11 traits for C set CSSLs in Shenzhen (SZ). P values are shown below and bolds mean P < 0.01. Table S8. Candidate genes in the 119 kb interval for qGL1. Bolds indicate differential expressed genes based on RNA-seq data. Table S9. Primers used for sequence identification. (DOCX 45 kb) [file 12284_2019_293_MOESM2_ESM.docx]

**Supplementary Table 1** Eleven traits of Nipponbare, PA64s and 9311 observed in Shengzhen (SZ) and Hangzhou (HZ)

| Traits | PA64s-SZ | 9311-SZ | NIP-SZ | PA64s-HZ | 9311-HZ | NIP-HZ |
| --- | --- | --- | --- | --- | --- | --- |
| PH (cm) | 75.1±2.5⁎ | 101.4±2.9⁎⁎ | 67.6±2.6 | 73.3±2.9 | 129±3.3⁎⁎ | 79.8±3.7 |
| PN | 10.7±0.5⁎⁎ | 7.3±0.5⁎⁎ | 16.7±1.2 | 16±0.8⁎⁎ | 7.3±0.5⁎⁎ | 12.7±0.5 |
| FLL (cm) | 29±1.08 | 27.3±0.74 | 27.34±3.39 | 24.43±0.39⁎⁎ | 28.47±0.37 | 28.6±0.83 |
| FLW (cm) | 1.72±0.09⁎ | 1.96±0.04⁎⁎ | 1.41±0.11 | 1.53±0.03⁎⁎ | 2.02±0.05⁎⁎ | 1.1±0.04 |
| PPB | 13.3±0.5⁎⁎ | 10.7±0.5⁎⁎ | 7.3±0.5 | 12.3±0.5⁎⁎ | 11.3±0.5⁎ | 9.7±0.5 |
| SPB | 40.4±2.9⁎⁎ | 32±2.4⁎⁎ | 15.3±2.1 | 33.3±2.9⁎⁎ | 45.7±2.9⁎⁎ | 11.3±1.2 |
| PL (cm) | 22.63±0.84⁎ | 23.17±0.29⁎⁎ | 18.62±0.94 | 20±0.41 | 23.27±0.29⁎⁎ | 20.23±0.57 |
| SN | 236.3±20.5⁎⁎ | 155±8.6⁎⁎ | 83.3±10.3 | 170.7±15.1⁎⁎ | 166.3±9⁎⁎ | 88.7±7.8 |
| GL (mm) | 8.71±0.16⁎⁎ | 9.87±0.19⁎⁎ | 7.43±0.29 | 8.74±0.08⁎⁎ | 9.87±0.17⁎⁎ | 6.92±0.2 |
| GW (mm) | 2.53±0.06⁎⁎ | 2.67±0.07⁎⁎ | 3.04±0.06 | 2.53±0.05⁎⁎ | 2.76±0.06⁎⁎ | 3.23±0.1 |
| TGW (g) | 18.15±0.53⁎⁎ | 32.84±0.29⁎⁎ | 27.07±0.33 | 18.74±0.25⁎⁎ | 31.05±0.2⁎⁎ | 21.62±0.2 |

Mean ± SD. (n=5).

* and ** indicate the least significant difference at 0.05 and 0.01 probability level compared with Nipponbare in SZ or HZ, respectively.

**Supplementary Table 2** Correlation analysis of 11 traits for A set CSSLs in Hangzhou (HZ)

|  | PH-HZ | PN-HZ | FLL-HZ | FLW-HZ | PPB-HZ | SPB-HZ | PL-HZ | SN-HZ | GL-HZ | GW-HZ |
| --- | --- | --- | --- | --- | --- | --- | --- | --- | --- | --- |
| PN-HZ | -0.2047 |  |  |  |  |  |  |  |  |  |
|  | 0.0550 |  |  |  |  |  |  |  |  |  |
| FLL-HZ | 0.0773 | -0.2573 |  |  |  |  |  |  |  |  |
|  | 0.8182 | 0.0780 |  |  |  |  |  |  |  |  |
| FLW-HZ | 0.1118 | -0.1712 | **0.4163** |  |  |  |  |  |  |  |
|  | 0.4528 | 0.4095 | **0.0008** |  |  |  |  |  |  |  |
| PPB-HZ | 0.1775 | -0.1697 | 0.1274 | 0.1070 |  |  |  |  |  |  |
|  | 0.1248 | 0.1275 | 0.3395 | 0.3787 |  |  |  |  |  |  |
| SPB-HZ | -0.1898 | -0.2762 | -0.2997 | 0.2926 | 0.1390 |  |  |  |  |  |
|  | 0.0856 | 0.0120 | 0.0249 | 0.0287 | 0.0230 |  |  |  |  |  |
| PL-HZ | **0.2294** | -0.0592 | 0.0245 | 0.6858 | 0.2109 | -0.1761 |  |  |  |  |
|  | **0.0030** | 0.5975 | 1.0517 | 0.5623 | 0.0572 | 0.1134 |  |  |  |  |
| SN-HZ | 0.2955 | -0.3331 | 0.1685 | 0.1516 | -0.0987 | **0.9036** | -0.0075 |  |  |  |
|  | 0.0540 | 0.2022 | 0.2209 | 0.3008 | 0.3779 | **<.0001** | 0.9469 |  |  |  |
| GL-HZ | **0.3171** | -0.0313 | -0.0868 | **0.6584** | 0.0879 | **-0.4282** | **0.4795** | **-0.3784** |  |  |
|  | **0.0039** | 0.7803 | 0.1372 | **0.0028** | 0.4321 | **<.0001** | **<.0001** | **0.0005** |  |  |
| GW-HZ | 0.0689 | 0.2215 | 0.1191 | **0.4703** | -0.0336 | **-0.3179** | 0.2186 | -0.3183 | **0.5961** |  |
|  | 0.7407 | 0.6477 | 0.3373 | **<.0001** | 0.7642 | **0.0036** | 0.0485 | 0.0336 | **<.0001** |  |
| TGW-HZ | 0.2451 | 0.0458 | 0.1298 | **0.3599** | 0.0185 | **-0.3896** | **0.4676** | **-0.3405** | **0.8495** | **0.7588** |
|  | 0.0539 | 0.3827 | 0.2986 | **0.0088** | 0.9398 | **0.0003** | **<.0001** | **0.0017** | **<.0001** | **<.0001** |

P values are shown below and bolds mean P < 0.01.

**Supplementary Table 3** Correlation analysis of 11 traits for A set CSSLs in Shenzhen (SZ)

|  | PH-SZ | PN-SZ | FLL-SZ | FLW-SZ | PPB-SZ | SPB-SZ | PL-SZ | SN-SZ | GL-SZ | GW-SZ |
| --- | --- | --- | --- | --- | --- | --- | --- | --- | --- | --- |
| PN-SZ | **-0.3187** |  |  |  |  |  |  |  |  |  |
|  | **0.0042** |  |  |  |  |  |  |  |  |  |
| FLL-SZ | -0.0696 | -0.2318 |  |  |  |  |  |  |  |  |
|  | 0.5421 | 0.0398 |  |  |  |  |  |  |  |  |
| FLW-SZ | 0.1007 | -0.1543 | **0.3751** |  |  |  |  |  |  |  |
|  | 0.3773 | 0.1746 | **0.0007** |  |  |  |  |  |  |  |
| PPB-SZ | 0.1812 | 0.0155 | 0.1048 | 0.1092 |  |  |  |  |  |  |
|  | 0.1124 | 0.9619 | 0.3613 | 0.3412 |  |  |  |  |  |  |
| SPB-SZ | -0.1985 | 0.1721 | 0.0717 | 0.0619 | 0.2542 |  |  |  |  |  |
|  | 0.0815 | 0.1319 | 0.5326 | 0.5901 | 0.0238 |  |  |  |  |  |
| PL-SZ | **0.1911** | 0.0382 | **0.3572** | 0.1068 | 0.1453 | 0.1422 |  |  |  |  |
|  | **0.0024** | 0.7398 | **0.0013** | 0.3523 | 0.2014 | 0.2114 |  |  |  |  |
| SN-SZ | -0.2206 | 0.1388 | 0.1946 | 0.0703 | **0.4161** | **0.7880** | **0.3080** |  |  |  |
|  | 0.0523 | 0.2254 | 0.0878 | 0.5407 | **0.0001** | **<.0001** | **0.0058** |  |  |  |
| GL-SZ | **0.2373** | -0.0970 | 0.1814 | **0.3426** | -0.1132 | **-0.3630** | 0.2517 | **-0.3552** |  |  |
|  | **0.0038** | 0.4014 | 0.1143 | **0.0023** | 0.3237 | **0.0011** | 0.0262 | **0.0014** |  |  |
| GW-SZ | -0.1153 | -0.0066 | 0.1073 | **0.4237** | -0.1745 | -0.0825 | 0.1414 | 0.0810 | **0.5247** |  |
|  | 0.3182 | 0.9548 | 0.3528 | **0.0001** | 0.1264 | 0.4730 | 0.2169 | 0.4810 | **<.0001** |  |
| TGW-SZ | 0.1506 | -0.1521 | 0.1170 | **0.3242** | -0.1738 | -0.2791 | 0.1952 | -0.2444 | **0.8147** | **0.5259** |
|  | 0.1910 | 0.1866 | 0.3110 | **0.0040** | 0.1280 | 0.0134 | 0.0868 | 0.0310 | **<.0001** | **<.0001** |

P values are shown below and bolds mean P < 0.01.

**Supplementary Table 4** Correlation analysis of 11 traits for B set CSSLs in Hangzhou (HZ)

|  | PH-HZ | PN-HZ | FLL-HZ | FLW-HZ | PPB-HZ | SPB-HZ | PL-HZ | SN-HZ | GL-HZ | GW-HZ |
| --- | --- | --- | --- | --- | --- | --- | --- | --- | --- | --- |
| PN-HZ | -0.2011 |  |  |  |  |  |  |  |  |  |
|  | 0.1409 |  |  |  |  |  |  |  |  |  |
| FLL-HZ | 0.1379 | -0.2025 |  |  |  |  |  |  |  |  |
|  | 0.3155 | 0.1381 |  |  |  |  |  |  |  |  |
| FLW-HZ | 0.2426 | -0.3210 | **0.3622** |  |  |  |  |  |  |  |
|  | 0.0744 | 0.0169 | **0.0066** |  |  |  |  |  |  |  |
| PPB-HZ | 0.0275 | -0.2695 | **0.4378** | 0.3184 |  |  |  |  |  |  |
|  | 0.8422 | 0.0467 | **0.0008** | 0.0178 |  |  |  |  |  |  |
| SPB-HZ | -0.1843 | -0.2102 | 0.3446 | 0.2704 | **0.4740** |  |  |  |  |  |
|  | 0.1780 | 0.1236 | 0.0200 | 0.0458 | **0.0003** |  |  |  |  |  |
| PL-HZ | **0.4360** | -0.1239 | **0.5639** | **0.4069** | 0.1068 | 0.0110 |  |  |  |  |
|  | **0.0009** | 0.3675 | **<.0001** | **0.0020** | 0.4379 | 0.9943 |  |  |  |  |
| SN-HZ | -0.0712 | **-0.3510** | **0.4660** | **0.3628** | **0.7170** | **0.8775** | 0.0701 |  |  |  |
|  | 0.6055 | **0.0086** | **0.0003** | **0.0045** | **<.0001** | **<.0001** | 0.6110 |  |  |  |
| GL-HZ | 0.1560 | 0.0450 | -0.0191 | 0.1782 | -0.0177 | -0.1064 | 0.2907 | -0.0529 |  |  |
|  | 0.2555 | 0.7444 | 0.8902 | 0.1931 | 0.8981 | -0.4393 | 0.0313 | 0.7013 |  |  |
| GW-HZ | -0.0161 | -0.0163 | -0.0459 | 0.0192 | -0.1059 | 0.0102 | 0.0637 | -0.0937 | -0.0229 |  |
|  | 0.9073 | 0.9063 | 0.7391 | 0.8896 | 0.4417 | 0.9413 | 0.6440 | 0.4963 | 0.8680 |  |
| TGW-HZ | 0.1074 | 0.0541 | -0.0916 | -0.0009 | -0.2859 | -0.2782 | 0.2405 | **-0.3546** | **0.4572** | **0.6801** |
|  | 0.4352 | 0.6948 | 0.5061 | 0.9951 | 0.0343 | 0.0397 | 0.0770 | **0.0079** | **0.0005** | **<.0001** |

P values are shown below and bolds mean P < 0.01.

**Supplementary Table 5** Correlation analysis of 11 traits for B set CSSLs in Shenzhen (SZ)

|  | PH-SZ | PN-SZ | FLL-SZ | FLW-SZ | PPB-SZ | SPB-SZ | PL-SZ | SN-SZ | GL-SZ | GW-SZ |
| --- | --- | --- | --- | --- | --- | --- | --- | --- | --- | --- |
| PN-SZ | -0.2697 |  |  |  |  |  |  |  |  |  |
|  | 0.0609 |  |  |  |  |  |  |  |  |  |
| FLL-SZ | 0.1113 | 0.1118 |  |  |  |  |  |  |  |  |
|  | 0.4464 | 0.4443 |  |  |  |  |  |  |  |  |
| FLW-SZ | -0.0093 | -0.0988 | 0.1977 |  |  |  |  |  |  |  |
|  | 0.9495 | 0.4995 | 0.1480 |  |  |  |  |  |  |  |
| PPB-SZ | -0.3020 | 0.2027 | 0.1159 | 0.1618 |  |  |  |  |  |  |
|  | 0.0350 | 0.1624 | 0.3995 | 0.2379 |  |  |  |  |  |  |
| SPB-SZ | -0.2878 | 0.2909 | -0.1168 | 0.1228 | **0.4145** |  |  |  |  |  |
|  | 0.0449 | 0.0426 | 0.3959 | 0.3717 | **0.0017** |  |  |  |  |  |
| PL-SZ | 0.2747 | -0.0453 | 0.2397 | -0.0019 | 0.0407 | -0.1890 |  |  |  |  |
|  | 0.0562 | 0.7572 | **0.0780** | 0.9890 | 0.7679 | 0.1671 |  |  |  |  |
| SN-SZ | -0.2765 | 0.2488 | **0.0068** | 0.1988 | **0.6821** | **0.8815** | -0.1033 |  |  |  |
|  | 0.0544 | 0.0848 | 0.9608 | 0.1458 | **<.0001** | **<.0001** | 0.4530 |  |  |  |
| GL-SZ | **0.3997** | -0.2066 | 0.1082 | 0.1356 | **-0.3725** | **-0.5648** | **0.4368** | **-0.5085** |  |  |
|  | **0.0044** | 0.1544 | 0.4315 | 0.3235 | **0.0051** | **<.0001** | **0.0009** | **<.0001** |  |  |
| GW-SZ | -0.1247 | 0.0295 | -0.1372 | -0.0185 | -0.1669 | 0.1475 | -0.1961 | -0.0109 | -0.1310 |  |
|  | 0.3931 | 0.8405 | 0.3180 | 0.8931 | 0.2232 | 0.2827 | 0.1513 | 0.9368 | 0.3405 |  |
| TGW-SZ | 0.3195 | -0.0546 | 0.0283 | 0.0880 | **-0.4143** | **-0.3450** | **0.4040** | **-0.4411** | **0.6472** | **0.2600** |
|  | 0.0252 | 0.7095 | 0.8376 | 0.5228 | **0.0017** | **0.0099** | **0.0022** | **0.0007** | **<.0001** | **0.0055** |

P values are shown below and bolds mean P < 0.01.

**Supplementary Table 6** Correlation analysis of 11 traits for C set CSSLs in Hangzhou (HZ)

|  | PH-HZ | PN-HZ | FLL-HZ | FLW-HZ | PPB-HZ | SPB-HZ | PL-HZ | SN-HZ | GL-HZ | GW-HZ |
| --- | --- | --- | --- | --- | --- | --- | --- | --- | --- | --- |
| PN-HZ | -0.0897 |  |  |  |  |  |  |  |  |  |
|  | 0.4808 |  |  |  |  |  |  |  |  |  |
| FLL-HZ | 0.1937 | -0.1450 |  |  |  |  |  |  |  |  |
|  | 0.1251 | 0.2530 |  |  |  |  |  |  |  |  |
| FLW-HZ | 0.2977 | -0.2200 | 0.1300 |  |  |  |  |  |  |  |
|  | 0.0169 | 0.0807 | 0.3059 |  |  |  |  |  |  |  |
| PPB-HZ | 0.2874 | 0.0562 | -0.3135 | 0.3061 |  |  |  |  |  |  |
|  | 0.0388 | 0.9818 | 0.0237 | 0.0273 |  |  |  |  |  |  |
| SPB-HZ | 0.2288 | -0.0091 | 0.0621 | **0.6178** | **0.5508** |  |  |  |  |  |
|  | 0.1027 | 0.9492 | 0.8764 | **<.0001** | **<.0001** |  |  |  |  |  |
| PL-HZ | **0.4098** | -0.0344 | 0.1518 | **0.4068** | 0.0597 | 0.3400 |  |  |  |  |
|  | **0.0008** | 0.7871 | 0.2311 | **0.0008** | 0.7801 | 0.0137 |  |  |  |  |
| SN-HZ | 0.2662 | 0.0195 | -0.0782 | **0.5931** | **0.7470** | **0.9357** | 0.2982 |  |  |  |
|  | 0.0565 | 0.9467 | 0.5815 | **<.0001** | **<.0001** | **<.0001** | 0.0318 |  |  |  |
| GL-HZ | 0.2642 | 0.1712 | 0.0602 | **0.3619** | 0.0878 | 0.2308 | 0.3105 | 0.2461 |  |  |
|  | 0.0349 | 0.1761 | 0.6363 | **0.0033** | 0.5361 | 0.0997 | 0.0125 | 0.0786 |  |  |
| GW-HZ | 0.0721 | -0.1219 | -0.0092 | -0.0592 | -0.2530 | -0.2150 | 0.1816 | -0.2635 | -0.2544 |  |
|  | 0.6673 | 0.4658 | 0.9563 | 0.7243 | 0.2029 | 0.2815 | 0.2753 | 0.1841 | 0.1233 |  |
| TGW-HZ | 0.2043 | 0.1747 | 0.0239 | -0.0483 | **-0.3604** | -0.2250 | 0.1732 | -0.3053 | **0.3538** | **0.5659** |
|  | 0.1055 | 0.1675 | 0.8513 | 0.7045 | **0.0087** | 0.1088 | 0.1711 | 0.0278 | **0.0041** | **0.0002** |

P values are shown below and bolds mean P < 0.01.

**Supplementary Table 7** Correlation analysis of 11 traits for C set CSSLs in Shenzhen (SZ)

|  | PH-SZ | PN-SZ | FLL-SZ | FLW-SZ | PPB-SZ | SPB-SZ | PL-SZ | SN-SZ | GL-SZ | GW-SZ |
| --- | --- | --- | --- | --- | --- | --- | --- | --- | --- | --- |
| PN-SZ | **-0.4591** |  |  |  |  |  |  |  |  |  |
|  | **0.0001** |  |  |  |  |  |  |  |  |  |
| FLL-SZ | **0.4132** | -0.2855 |  |  |  |  |  |  |  |  |
|  | **0.0006** | 0.0202 |  |  |  |  |  |  |  |  |
| FLW-SZ | 0.2720 | -0.4116 | **0.4047** |  |  |  |  |  |  |  |
|  | 0.0272 | 0.0506 | **0.0008** |  |  |  |  |  |  |  |
| PPB-SZ | 0.2484 | -0.2857 | 0.0654 | 0.1964 |  |  |  |  |  |  |
|  | 0.0443 | 0.0200 | 0.6017 | 0.1141 |  |  |  |  |  |  |
| SPB-SZ | 0.3045 | -0.3106 | 0.1728 | 0.2020 | **0.6502** |  |  |  |  |  |
|  | 0.0129 | 0.0111 | 0.1652 | 0.1039 | **<.0001** |  |  |  |  |  |
| PL-SZ | **0.6468** | **-0.4281** | **0.4149** | **0.3656** | **0.4473** | **0.5231** |  |  |  |  |
|  | **<.0001** | **0.0003** | **0.0005** | **0.0025** | **0.0002** | **<.0001** |  |  |  |  |
| SN-SZ | 0.3104 | **-0.3661** | 0.1850 | 0.2587 | **0.7612** | **0.9436** | **0.5871** |  |  |  |
|  | 0.0112 | **0.0025** | 0.1370 | 0.0360 | **<.0001** | **<.0001** | **<.0001** |  |  |  |
| GL-SZ | **0.4620** | -0.3127 | 0.1694 | 0.2844 | 0.0800 | 0.0849 | 0.3008 | 0.1682 |  |  |
|  | **<.0001** | 0.0106 | 0.1738 | 0.0207 | 0.5233 | 0.4979 | 0.0141 | 0.1771 |  |  |
| GW-SZ | 0.0699 | -0.0171 | 0.0652 | 0.2170 | -0.2511 | -0.2491 | -0.0137 | -0.2450 | -0.0344 |  |
|  | 0.5771 | 0.8917 | 0.8408 | 0.0801 | 0.0420 | 0.0437 | 0.9128 | 0.0474 | 0.7841 |  |
| TGW-SZ | **0.3591** | -0.1983 | 0.2033 | 0.2327 | **-0.3750** | **-0.3760** | 0.1523 | **-0.3552** | **0.3738** | **0.6181** |
|  | **0.0031** | 0.1105 | 0.1017 | 0.0601 | **0.0019** | **0.0019** | 0.2221 | **0.0034** | **0.0020** | **<.0001** |

P values are shown below and bolds mean P < 0.01.

**Supplementary Table 8** Candidate genes in the 119 kb interval for *qGL1*

| Gene ID | Annotations |
| --- | --- |
| LOC_Os01g53079 | ubiquitin domain-containing protein 1 |
| LOC_Os01g53090 | pathogen-related protein |
| LOC_Os01g53100 | ubiquitin family protein |
| LOC_Os01g53130 | zinc finger C3HC4 type domain containing protein |
| **LOC_Os01g53140** | expressed protein |
| LOC_Os01g53150 | GTPase of unknown function domain containing protein |
| LOC_Os01g53160 | DUF623 domain containing protein |
| LOC_Os01g53170 | retrotransposon protein |
| LOC_Os01g53180 | retrotransposon protein |
| LOC_Os01g53190 | expressed protein |
| LOC_Os01g53200 | flavin monooxygenase |
| LOC_Os01g53210 | metal transporter Nramp3 |
| **LOC_Os01g53220** | HSF-type DNA-binding domain containing protein |
| LOC_Os01g53230 | expressed protein |
| LOC_Os01g53240 | BURP domain containing protein |
| **LOC_Os01g53250** | NADPH reductase |
| LOC_Os01g53255 | expressed protein |
| LOC_Os01g53260 | WRKY23 expressed |

Bolds indicate differential expressed genes based on RNA-seq data.

**Supplementary Table 9** Primers used for sequence identification

| **Name** | **Primer sequence** |
| --- | --- |
| Lax1F | CACTTACAAGATTGGCCATGACG |
| Lax1R | TGATATACTTGCCCGCGTGAATA |
| sd1F | CGCTCACTTCTCATCTCCAATCT |
| sd1R | AGGAGAACAGAGGAGAGGAGAAT |
| Nal1F | ATGAAGCCTTCGGACGATAAG |
| Nal1R | TCATTTCTCCAGGTCAAGGCTT |
| GW5F | CGTCTTGCAACCAACGCCGATGTTATAC |
| GW5R | GAGCGTGTGTAGGGAAGGAGCTGCATGA |
| RT140F | GGTGGATCGAGGAGTGGAAC |
| RT140R | GAATTCACGGACGATGCCCT |
| RT220F | GCAGCTCAACACCTACGGATTC |
| RT220R | TTCTTCTTCTTGCGCACGATCC |
| RT250F | TGTTAGCATAGTGTCATGGCGT |
| RT250R | GGATGTGGAGGAGGCAAAGAA |
| *OsActin1*F | CCATTGGTGCTGAGCGTTT |
| *OsActin1R* | CGCAGCTTCCATTCCTATGAA |
