# Supplementary figures and images for: Development of Three Sets of High-Throughput Genotyped Rice Chromosome Segment Substitution Lines and QTL Mapping for Eleven Traits
Source: Rice (N Y). 2019 May 10;12:33. doi: 10.1186/s12284-019-0293-y (PMC6510774; doi:10.1186/s12284-019-0293-y)

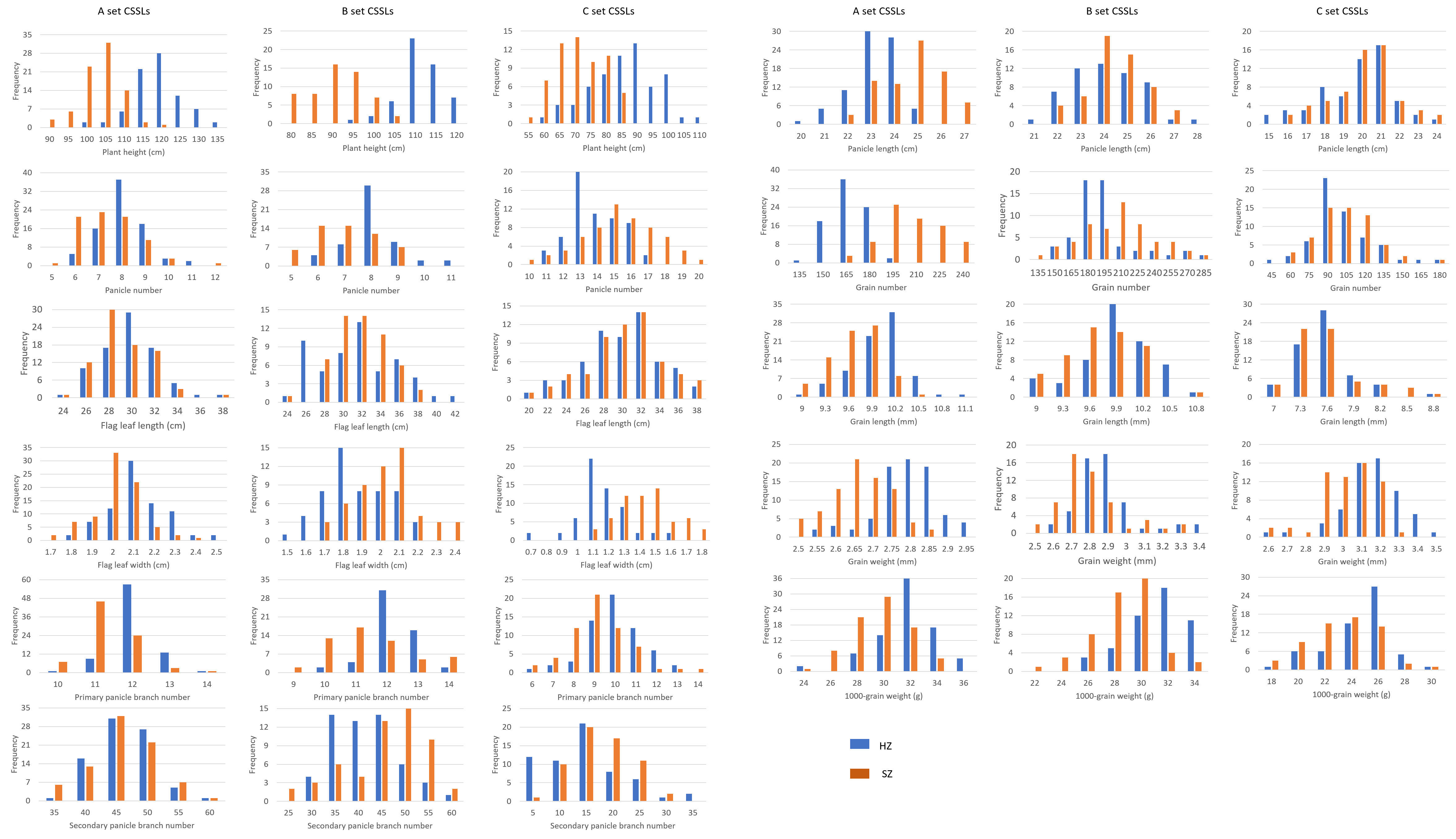

Supplement: Supplementary file 3 — Figure S1. Variation of the phenotypic traits in 3 sets of CSSLs. (TIF 1322 kb) [file 12284_2019_293_MOESM3_ESM.tif]

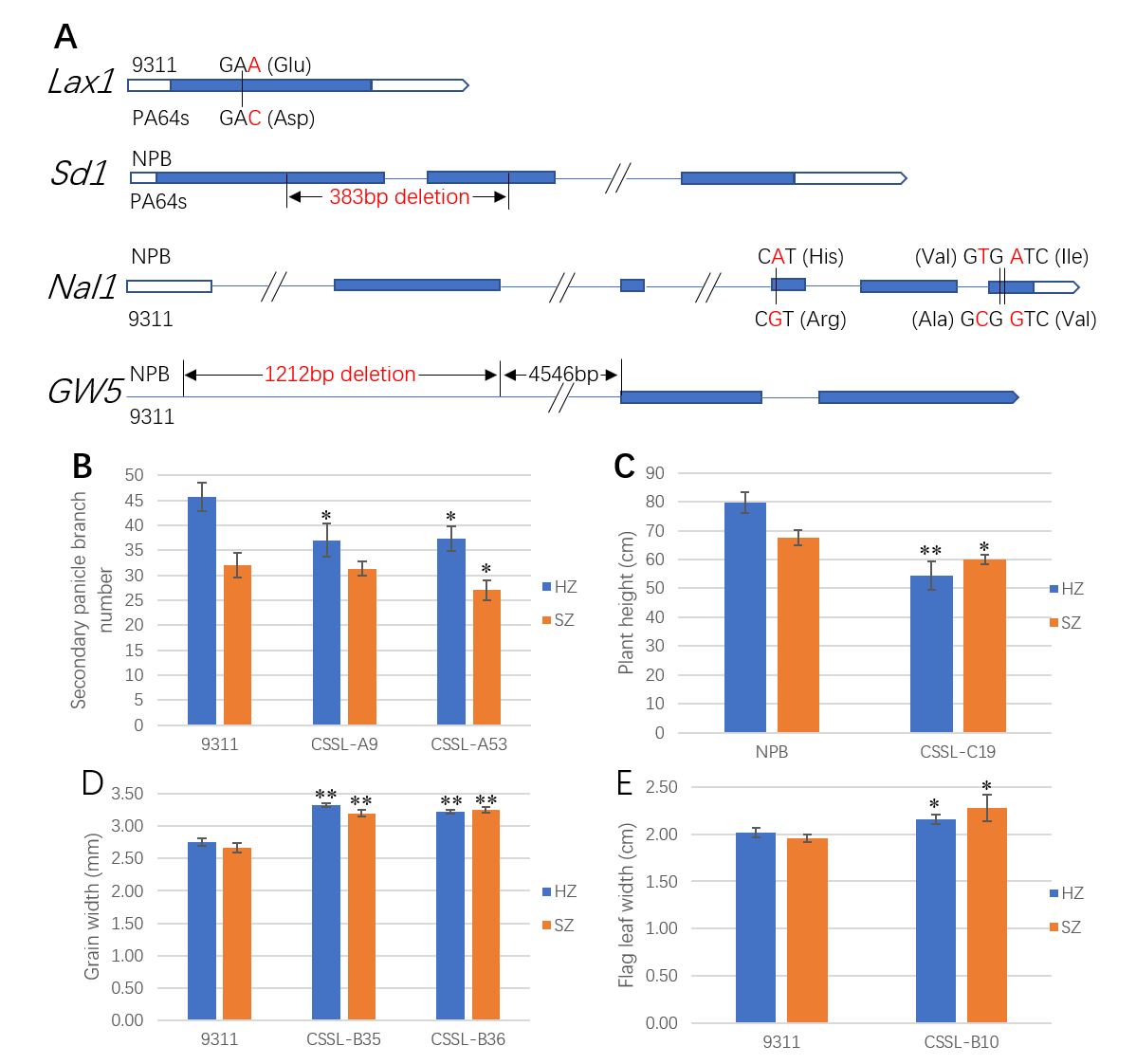

Supplement: Supplementary file 4 — Figure S2. Sequence difference and phenotypic variations on four reported genes. A. Difference sites between the related two parents. The difference sites are shown in red. Amino acids variations are listed in parenthesis. Empty blocks and blue blocks represent non-coding regions and exons, respectively. B-E. Phenotypic variation between CSSLs individuals and their recurrent parents. B. CSSL-A9 and CSSL-A53 harbor Lax1 of PA64s allele at qSPB1–2 locus. C. CSSL-C19 harbors Sd1 of PA64s allele at qPH1–2 locus. D. CSSL-B35 and CSSL-B36 harbor GW5 of Nipponbare allele at qGW5 locus. E. CSSL-B10 harbors Nal1 of Nipponbare allele at qFLW4 locus. Error bars are s.d. * and ** indicate the least significant difference at 0.05 and 0.01 probability level compared with the recurrent parent in SZ or HZ, respectively. NPB represents Nipponbare. (TIF 211 kb) [file 12284_2019_293_MOESM4_ESM.tif]

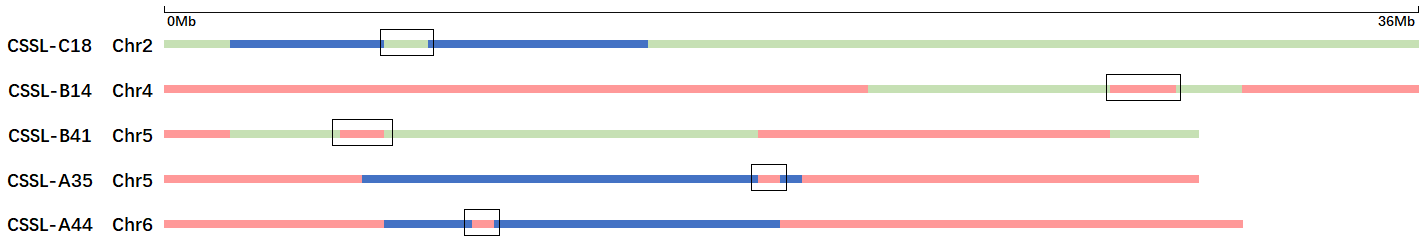

Supplement: Supplementary file 5 — Figure S3. Double-crossovers in CSSLs. Different colors represent different genotypes: red, 9311; blue, PA64s; green, Nipponbare. Double-crossovers were shown in black panes. (TIF 1447 kb) [file 12284_2019_293_MOESM5_ESM.tif]

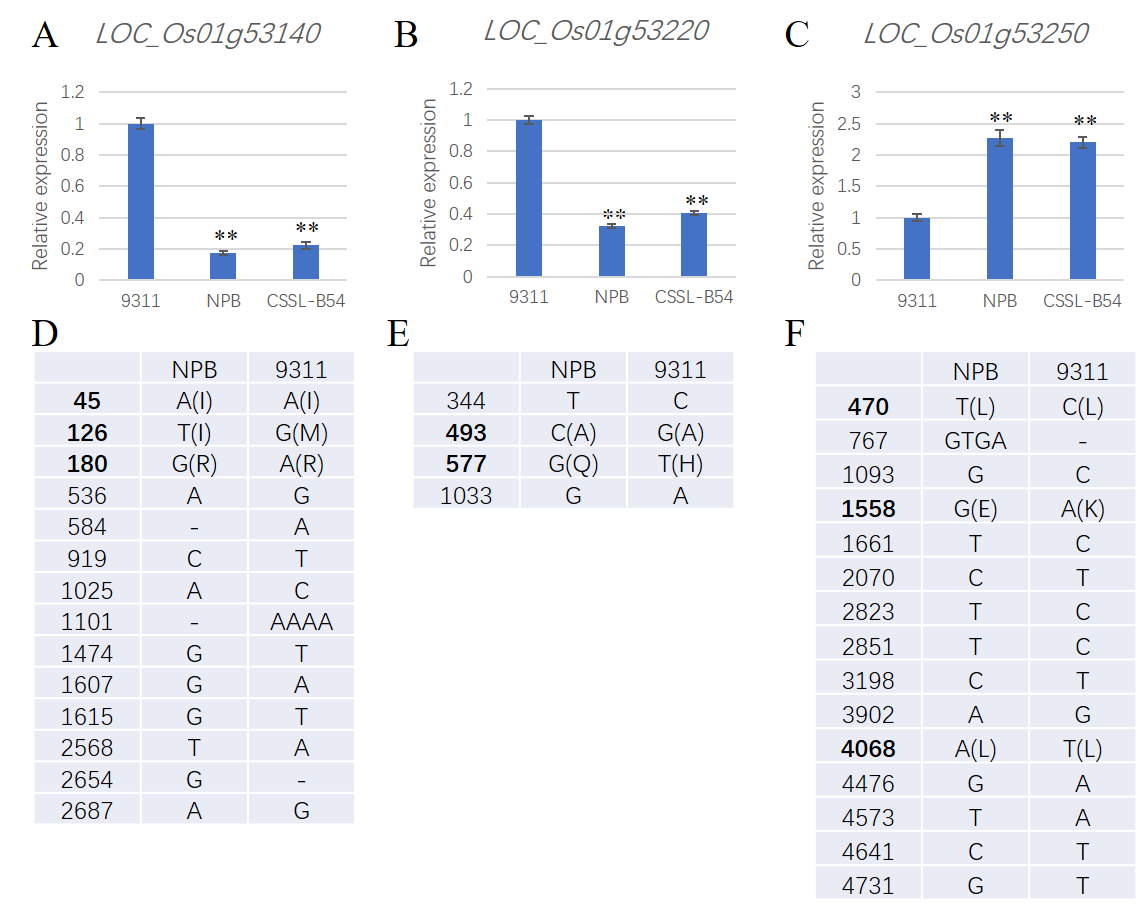

Supplement: Supplementary file 6 — Figure S4. Quantitative real-time RT-PCR analysis and Sequence variations of predicted genes. A-C.RNA relative expression level of 9311, Nip and CSSL-B54 in young panicle for LOC_Os01g53140, LOC_Os01g53140 and LOC_Os01g53250, respectively. D-F. Sequence variations between Nip and 9311 for LOC_Os01g53140, LOC_Os01g53140 and LOC_Os01g53250, respectively. Values represent means ± SD of three independent assays. ** indicate the least significant difference at 0.01 probability level compared with 9311. NPB represents Nipponbare. SNPs on the exons are shown in bold and Amino acids variations are listed in parenthesis. (TIF 3099 kb) [file 12284_2019_293_MOESM6_ESM.tif]
